# Proteomic changes associated with deletion of the *Magnaporthe oryzae *conidial morphology-regulating gene *COM1*

**DOI:** 10.1186/1745-6150-5-61

**Published:** 2010-11-02

**Authors:** Vijai Bhadauria, Li-Xia Wang, You-Liang Peng

**Affiliations:** 1 State Key Laboratory of Agrobiotechnology and MOA Key Laboratory of Molecular Plant Pathology, China Agricultural University, Beijing 100193, China, PR; 2 National Plant Gene Research Center, Beijing 100193, China, PR; 3 Current Address: Department of Biology, University of Saskatchewan, 112 Science place, Saskatoon, SK S7N 5E2, Canada

## Abstract

**Background:**

The rice blast disease caused by *Magnaporthe oryzae *is a major constraint on world rice production. The conidia produced by this fungal pathogen are the main source of disease dissemination. The morphology of conidia may be a critical factor in the spore dispersal and virulence of *M. oryzae *in the field. Deletion of a conidial morphology regulating gene encoding putative transcriptional regulator COM1 in *M. oryzae *resulted in aberrant conidial shape, reduced conidiation and attenuated virulence.

**Results:**

In this study, a two-dimensional gel electrophoresis/matrix assisted laser desorption ionization- time of flight mass spectrometry (2-DE/MALDI-TOF MS) based proteomics approach was employed to identify the cellular and molecular components regulated by the COM1 protein (COM1p) that might contribute to the aberrant phenotypes in *M. oryzae*. By comparing the conidial proteomes of *COM1 *deletion mutant and its isogenic wild-type strain P131, we identified a potpourri of 31 proteins that exhibited statistically significant alterations in their abundance levels. Of these differentially regulated proteins, the abundance levels of nine proteins were elevated and twelve were reduced in the Δ*com1 *mutant. Three proteins were detected only in the Δ*com1 *conidial proteome, whereas seven proteins were apparently undetectable. The data obtained in the study suggest that the COM1p plays a key role in transcriptional reprogramming of genes implicated in melanin biosynthesis, carbon and energy metabolism, structural organization of cell, lipid metabolism, amino acid metabolism, etc. Semi-quantitative RT-PCR analysis revealed the down-regulation of genes encoding enzymes involved in melanin biosynthesis in the *COM1 *mutant.

**Conclusions:**

Our results suggest that the COM1p may regulate the transcription of genes involved in various cellular processes indispensable for conidial development and appressorial penetration. These functions are likely to contribute to the effects of COM1p upon the aberrant phenotypes of *M. oryzae*.

**Reviewers:**

This article is reviewed by George V. Shpakovski, Karthikeyan Sivaraman (nominated by M. Madan Babu) and Lakshminarayan M. Iyer.

## Background

*Magnaporthe oryzae *is a heterothallic, haploid ascomycete fungus that causes rice blast, one of the most devastating diseases of rice (*Oryza sativa *L.) worldwide [1]. This fungal phytopathogen employs a bi-phasic 'stealth' hemibiotrophic infection strategy to colonize the host plants. The blast infection is initiated by the attachment of conidia to leaf surface. The conidia germinate immediately and germ tubes differentiate into specialized infection structures called appressoria [2]. After the appressoria have been formed, their internal turgor pressure increases massively. This facilitates the piercing of the rice cuticle and cell wall by the infection pegs that then expand into epidermal apoplastic space and differentiate into the biotrophic bulbous invasive hyphae. These hyphae then invaginate the host plasma membrane and become sealed into extra-invasive hyphal membrane compartment [3] similar to the extra-haustorial matrix developed by obligate biotrophic pathogens. Thereafter, the pathogen switches to the necrotrophic phase, associated with production of thin secondary hyphae that ramify intra- and inter-cellularly, killing and macerating the host tissues ahead of infection. At this stage, necrotic disease lesions become evident. The appearance of lesions is accompanied by the development of aerial conidiophores. The conidia of *M. oryzae *are sympodially arrayed at the tips of conidiophores [4]. The conidia are then dispersed by rain splashes or wind to reinitiate the disease cycle. Considering the role of conidia in infection cycle, the morphology of conidia may be a decisive factor in the dissemination of blast disease and initiation of plant infection.

*M. oryzae *produces three-celled pyriform conidia that are the primary inoculum and the main source for initiation and dissemination of the blast disease in field. To date, several mutants with defective conidiogenesis and altered conidial morphology, including *smo, con1, con2, con4, con7, acr1, chm1*, and *com1*, have been identified by chemical or insertional mutagenesis [5-9]. For instance, the Smo mutation (**S**pore **Mo**rphology) causes abnormally shaped conidia, which show a non-polarized shape. The Δ*smo *mutant occasionally produces conidia with two apical cells, as well as conidia that appear to lack apical cell completely. The *smo *mutant exhibits reduced virulence due to the production of misshapen appressoria [5,10]. Similarly, the conidia of *con *mutants show aberrant morphologies viz., terminally elongated conidia of the Δ*con1 *mutant and non-septate or two-celled conidia of the Δ*con2 *[6].

The gene *COM1 *(***Co****nidial ****M****orphology ****1***) encodes a nuclear localized protein that plays a critical role in maintaining the normal conidial shape in *M. oryzae*. The COM1p contains a putative ELL (RNA polymerase II elongation factor) domain (PMID: 17150956) (Additional file 1), which may confer the transcription regulator activity. The *COM1 *homologs are well distributed in other filamentous ascomycetes and basidiomycetes, including *Chaetomium globosum, Neurospora crassa, Fusarium graminearum, Aspergillus fumigatus, A. nidulans, Saccharomyces pombe *and *Cryptococcus neoformans*. So far, none of them has been functionally characterized.

**Figure 1:**
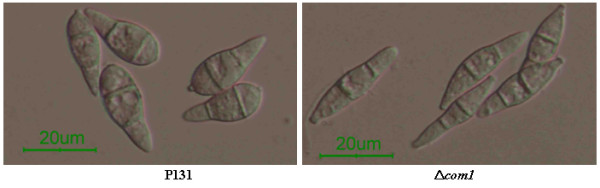
**Effect of *COM1 *deletion on conidial morphology**. Conidia of *M. oryzae *wild-type strain P131 (A) and conidia of Δ*com1 *mutant strain (B).

Deletion of the *COM1 *in *M. oryzae *wild-type isolate P131 background had resulted in pleiotropic phenotypical changes, such as altered conidial morphology, reduced conidiation, lightly melanized appressoria, suppression of penetration peg and infection hypha formation, and attenuated virulence. The conidia produced by the Δ*com1 *mutant were 25% longer and had narrower basal and middle cells than that of the P131 (Figure 1). Therefore, it is expected that the COM1p is implicated in regulating conidial development and normal conidium morphology in *M. oryzae*. The conidiation rate in the Δ*com1 *mutant was reduced to >4 folds than that of the strain P131 [9]. But it remains ambiguous how the COM1p regulates the conidium morphology.

We expect that the cellular and molecular components regulating the conidium morphology would be unveiled by proteomics as proteins are directly related to function (phenotype). Therefore, we exploited the proteomics approach with the aim to investigate the cellular and molecular basis of COM1-regulated phenotypes. Studies conducted to date have investigated the molecular mechanisms underlying compatible and non-compatible host-pathogen interactions and fungal development, including spore, spore germination and appressorial morphogenesis. The conidiospore proteome profiling of barley powdery mildew pathogen (*Blumeria graminis *f.sp. *hordei*) revealed that the majority of identified proteins had a predicted function in lipid, carbohydrate or protein metabolism [11]. Another study has shown that high energy production and accumulation of histone proteins are required during the germination of asexual uredospores of rust fungus, *Uromyces appendiculatus *[12]. Kim and colleagues [13] identified 5 induced proteins [two 20 S proteasome α subunits, scytalone dehydratase (SCD), serine carboxypeptidase Y (CPY) and an unknown protein] from the appressorial forming conidia of *M. oryzae*. The proteasome subunits may be involved in mobilizing storage proteins, whereas the SCD is a key enzyme of melanin biosynthesis and its role in appressorial penetration is well documented. The CPY is localized in vacuoles that play an important role in the maintaining of cellular homeostasis. Current proteomics methodologies using both 2-DE and MS provide a snapshot of the proteins expressed by an organism at the particular point in time when the organism is processed for protein purification and analysis.

In this study, a 2-DE/MALDI-TOF MS based proteomics approach was employed to catalogue proteins regulated by the COM1p. A repertoire of 31 proteins was found to be differentially regulated in the conidial proteome of Δ*com1 *mutant while comparing with an isogenic wild-type strain of *M. oryzae *P131. The functions of these proteins suggest that COM1p regulates a number of cellular and molecular components, including melanin biosynthesis, energy and carbon metabolism, structural organization, lipid metabolism, amino acid metabolism, etc. The data obtained in this study will be instrumental in deciphering mechanisms underlying the conidial morphology regulation.

## Results

### Differential abundance pattern display of the *M. oryzae *conidial proteomes of wild-type strain P131 and Δ*com1 *mutant

A quantitative comparison of the conidial proteomes of strain P131 and Δ*com1 *mutant was performed to investigate the effect of conidial morphology-regulating gene *COM1 *deletion in *M. oryzae*. To obtain the global profiles of both proteomes, we resolved conidial proteomes on two immobilized linear pH gradients (3-10 and 4-7) to cover both acidic and basic proteins. We found that the proteins with less than 20 KDa were out of gels after staining; therefore 12.5% polyacrylamide SDS gels were used in the second dimension to visualize these low molecular weight proteins.

**Figure 2:**
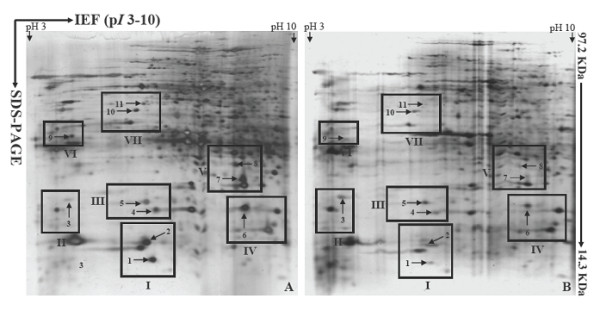
**Protein abundance profiles of samples extracted from conidia of *M. oryzae *field isolate P131 (A) and Δ*com1 *mutant (B)**. Proteins were separated by 2-DE. In the first dimension (IEF), 200 μg of total protein were loaded on 18 cm IPG strips with a linear pH gradient of 3-10. In the second dimension, 12.5% SDS-PAGE gels were used. Proteins were visualized using silver staining. Reproducible differentially expressed proteins were located in the framed areas (I to VII). A total of 11 proteins were identified as differentially regulated proteins.

**Figure 3:**
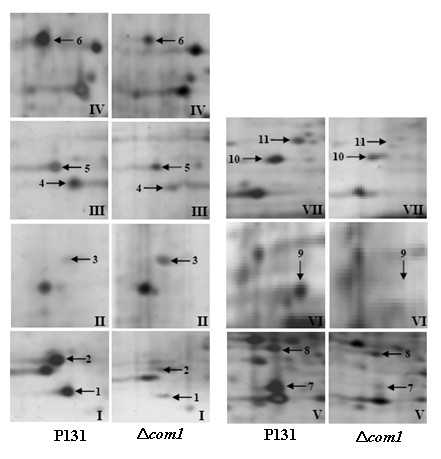
**Magnification views of the same regions of the gels framed in figure 2**.

Figure 2 shows two highly reproducible 2-DE gel images displaying conidial proteomes (on pH scale 3-10) of the strain P131 (Figure 2A) and the Δ*com1 *mutant (Figure 2B). Over 650 protein spots with *M*r between 14.4 and 97 KDa and a p*I *ranging from 3 to 10 were resolved on both gels. Reproducible differences in protein abundance patterns between the conidial proteomes of strain P131 and Δ*com1 *mutant were noticeable in 7 regions of the gels, designated frames I, II, III, IV, V, VI, and VII. Magnification views of all 7 frames are illustrated in Figure 3. Close-up views of frames revealed that eleven proteins had exhibited consistent and reproducible differential abundance patterns. Two of them (spots 1 and 2) were displayed in frame I, one (spot 3) in frame II, two (spots 4 and 5) in frame III, one (spot 6) in frame IV, two (spots 7 and 8) in frame V, one (spot 9) in frame VI, and two (spots 10 and 11) in frame VII. Of the 11 differentially regulated proteins, ten proteins (spots 1, 2, 4, 5, 6, 7, 8, 9, 10 and 11) were down-regulated and only one protein (spot 3) had shown induction in the Δ*com1 *mutant. Abundance histograms were made for all differentially regulated proteins by ImageMaster 2D Platinum software version 5.0 using % intensity (Additional file 2) of spots. The histogram analysis elucidated that ten proteins showed 2 or over 2-fold down-regulation in the proteome profile of Δ*com1 *(spot 1- 8.4, spot 2- 6.7, spot 4- 4.0, spot 5- 1.9, spot 6- 2.9, spot 7- 5.9, spot 8- 3.5, spot 9- 2.1, spot 10- 2.1 and spot 11- 5.4). Only one protein (spot 3) had shown 2.2-fold up-regulation in the Δ*com1 *mutant.

**Figure 4:**
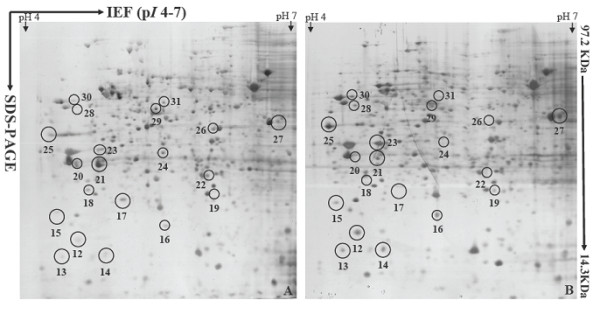
**Protein abundance profiles of samples extracted from conidia of *M. Oryzae *wild-type strain P131 (A) and Δ*com1 *mutant (B)**. Proteins were separated by 2-DE. In the first dimension (IEF), 200 μg of total protein were loaded on 18 cm IPG strips with a linear pH gradient of 4-7. In the second dimension, 12.5% SDS-PAGE gels were used. Proteins were visualized using silver staining.

**Figure 5:**
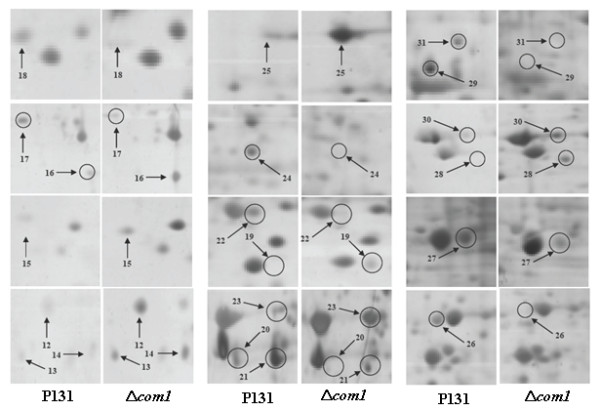
**Close-up views of protein spots which were differentially regulated on pH scale 4-7**.

Figure 4 depicts two best represented 2-DE gels showing conidial proteomes (on pH scale 4-7) of the strain P131 (Figure 4A) and the Δ*com1 *mutant (Figure 4B). Around 700 protein spots with *M*r between 14.4 and 97 KDa and a p*I *ranging from 4 to 7 were detected on both gels after a basic spot analysis by ImageMaster 2D Platinum software. This number was reduced to approximately 600 by ignoring very faint spots, spots with undefined shapes and areas where electrophoresis caused irregularity in the spot pattern. A number of proteins with differential abundance levels could then be detected in the conidial proteome of Δ*com1 *mutant. After manual extraction and statistical analysis of the corresponding data, significant results were selected. Altogether, 20 protein spots were found to be differentially regulated. Figure 5 represents close-up views of all 20 protein spots. Among 20 reproducible differentially regulated proteins, eight (spots 12, 13, 14, 16, 23, 25, 27 and 30) were found to be induced and only 2 (spots 21 and 29) had shown reduction. Interestingly, three proteins (spots 15, 19 and 28) were found only in the conidial proteome of Δ*com1 *mutant whereas seven proteins (spots 17, 18, 20, 22, 24, 26 and 31) had not detected in the Δ*com1 *mutant. Abundance histogram analysis revealed that four proteins (spot 12- 9.3, spot 13- 15.8, spot 14- 11.7 and spot 16- 5.9) showed over 5-fold induction in the Δ*com1 *mutant. Rest of induced proteins (spot 23- 1.7, spot 25- 2.4, spot 27- 1.97, and spot 30- 1.47) displayed <3-fold expression. Spots 21 and 29 had exhibited 6.67-fold and 1.82-fold down-regulation, respectively in the Δ*com1 *conidial proteome (Additional file 2).

### Identification of proteins regulated in response to *M. oryzae COM1 *deletion by MALDI-TOF MS

**Figure 6:**
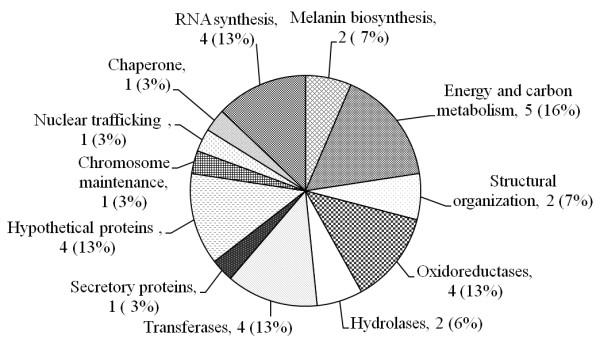
**Cellular and molecular components regulated by *COM1***.

All 31 differentially regulated proteins were analyzed by MS using MALDI as an ion source and TOF as mass analyzer, and the best homolog for each protein is listed in the table 1 (see also additional file 3). Among 31 proteins, 21 were identified as proteins with known functions or significant homology to known proteins. Homology search and sequence analyses of identified proteins revealed the identification of 12 potential groups of proteins regulated by the COM1p: melanin biosynthesis, energy and carbon metabolism, oxidoreductases, hydrolases, transferases, secretory proteins, chromosome maintenance protein, nuclear trafficking protein, molecular chaperone, RNA synthesis related proteins, and hypothetical proteins (Figure 6).

**Table 1:** Differentially regulated proteins (on pH scales pH 3-10 and pH 4-7) identified by MALDI-TOF MS.

**Spot**^**A**^	**Accession no.**^**B**^	Protein name	**Mr (KDa)/pI**^**C**^	**Hit ratio**^**D**^	Mascot score	**Sequence Coverage %**^**E**^
1	gi|39940362	Scytalone dehydratase	20.2/6.25	6/9	80	52
2	gi|39971339	Actin related protein -2	44.7/6.2	16/19	166	51
3	gi|58257439	Conserved hypothetical protein MGG_02610.5	37.7/4.76	9/10	125	50
4	gi|3287916	Tetrahydroxynaphthalene reductase	30/6.67	18/20	202	72
5	gi|39973539	Glyceraldehyde-3-phosphate dehydrogenase	35.9/6.91	13/14	171	64
6	gi|39960255	Malate dehydrogenase	35.1/8.31	16/16	214	62
7	gi|145608532	Glutaryl-CoA dehydrogenase	46/8.37	13/15	156	53
8	gi|39976425	Predicted protein MGG_05861.5	42.8/7.79	19/22	182	59
9	gi|39974499	Beta-tubulin	49.9/4.51	11/12	126	36
10	gi|145608354	ATP synthase beta chain, mitochondrial	55.8/5.27	15/17	162	47
11	gi|145609776	Hypothetical protein MGG_02992.5	53.3/6.31	14/18	124	41
12	gi|145602787	Hypothetical protein MGG_09874.5	22.9/4.98	8/9	120	68
13	gi|39944616	Predicted protein MGG_04319.5	16.8/4.79	4/5	69	52
14	gi|39968283	Hypothetical protein MGG_02234.5	14.8/4.83	3/3	46	19
15	gi|39946616	SMN family protein Smn1 [*Schizosaccharomyces pombe*]	18.3/4.74	6/6	103	60
16	gi|39975841	Molybdopterin binding domain protein	32.8/5.50	9/10	109	44
17	gi|7051772	Ran GTPase binding protein Mog1 [*Schizosaccharomyces japonicus*]	21.8/4.48	7/10	92	55
18	gi|39943944	Hsp70 nucleotide exchange factor fes1	23.4/4.26	4/5	52	22
19	gi|39976891	Adenylylsulfate kinase	23.2/6.69	6/8	75	45
20	gi|39970057	Predicted protein MGG_10637.5	36.1/4.87	12/14	144	54
21	gi|145601361	ATP25 protein [*Saccharomyces cerevisiae*]	31.2/4.95	7/9	92	45
22	gi|145604182	Hypothetical protein MGG_10674.5	44.9/6.66	13/16	141	44
23	gi|39971327	Hypothetical protein MGG_10684.5	37.2/4.85	9/11	106	37
24	gi|145616429	Hypothetical protein MGG_07401.5	39.3/5.08	11/12	140	49
25	gi|145603364	Serine/threonine protein phosphatase PP2A catalytic subunit	36.8/4.59	14/14	195	67
26	gi|39945404	Cytochrome P450 phenylacetate 2-hydroxylase	58.3/6.52	19/20	194	50
27	gi|39970147	Isovaleryl-CoA dehydrogenase-like protein	50.4/7.0	13/18	125	44
28	gi|8102490	Cell cycle control protein (Cwf8), putative [*Talaromyces stipitatus*].	51.7/4.95	12/14	126	36
29	gi|145614390	Vacuolar ATP synthase subunit H	53.6/5.04	18/22	179	55
30	gi|145613273	DNA replication licensing factor mcm3 [*Verticillium albo-atrum*]	58.4/4.84	17/22	151	45
31	gi|240110283	DHHC zinc finger domain containing protein [*Coccidioides posadasii*]	56.4/5.41	12/13	143	42

PMF data were utilized for protein identification by analyzing the size of tryptic fragments via the MASCOT (Matrix Science) search engine using the entire NCBI fungal protein database.

A) Spot: the numbers of proteins on gels.

B) Accession no.: the number of the predicted protein in NCBI-NR protein database.

C) Calculated from the *M. grisea *70-15 sequence using the ExPASy Compute pI/Mw tool.

D) Hit ratio: Number of peptide masses matched/number of masses submitted to the peptide mass fingerprint database search.

E) Per cent of protein sequence covered by matched peptides (PMF).

**Figure 7:**
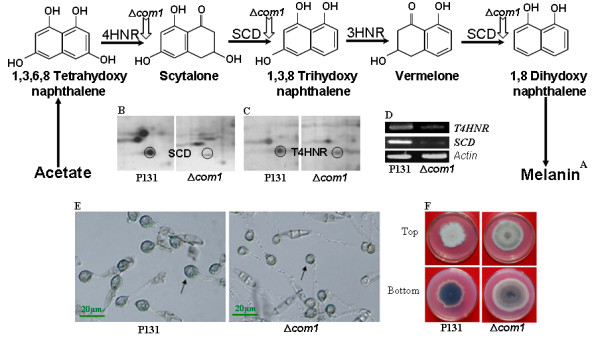
**The effect of *COM1* deletion on melanin biosynthetic pathway**. Fungal melanin biosynthetic pathway (A); close-up views of 2D gels comprising protein spots 1 (SCD) and 4 (T4HNR) (B-C); RT-PCR analysis of genes encoding SCD and T4HNR (D); appressoria forming conidia of wild-type strain P131 and Δ*com1 *mutants on inductive surface (E); and top and bottom views of fungal colonies grown on complete medium under nitrogen starvation (F).

The proteins down-regulated in the Δ*com1 *conidial proteome are mainly involved in melanin biosynthesis (spots 1 and 4), structural framework (spots 2 and 9), carbon and energy metabolism (spots 5, 6, 10, 21 and 29), and amino acid metabolism (spot 7). Spots 1 and 4 were identified as SCD and tetrahydroxynaphthalene reductase (4HNR), respectively. Both are key enzymes of the melanin biosynthetic pathway (Figure 7A). Spots 5, 6, 10, 21 and 29 were resembled with glyceraldehyde-3-phosphate dehydrogenase (GAPDH), mitochondrial malate dehydrogenase (m-MDH), β-subunit of mitochondrial ATP-synthase (m-ATPase), ATP25 and H-subunit of vacuolar H^+^-ATP synthase (v-ATPase), respectively. Both dehydrogenase enzymes are responsible for producing reducing equivalents (NADH) during cellular respiration while m- and v-ATP synthase are required for genrarating energy currency and acidification of subcellular organelles, respectively. Two proteins implicated in cellular structural organization (spot 2 as to actin related protein 2 [ARP2] and spot 9 as beta-tubulin) were identified as down-regulated protein in the conidial proteome of the Δ*com1 *mutant.

Four differentially regulated oxidoreductases or related proteins (spots 7, 16, 26 and 27) were also picked up by the comparative proteomic analysis of P131 and Δ*com1 *strains. The peptide mass fingerprints (PMFs) obtained from spots 7 and 27 were matched to two acyl-CoA dehydrogenases, glutaryl-and isovaleryl-CoA dehydrogenase (GcdH and IVD), respectively. GcdH is active in lysine metabolism whereas IVD catalyzes the third step in leucine metabolism. In addition to acyl-CoA dehydrogenase, we also identified a molybdopterin binding domain protein (spot 16), which shows cofactor binding activity and phenylacetate 2-hydroxylase (spot 26), involved in the phenylacetate metabolism.

We identified two hydrolases (spots 3 and 25) in the conidial proteomes. Spot 25 was identified as a catalytic subunit of protein phosphatase 2A (PP2A) that induced in the Δ*com1 *mutant. Protein phosphatases remove phosphate group from amino acids like serine, threonine and tyrosine. Dephosphorylation of proteins is a versatile mechanism for regulating the activity of enzymes [14].

Analysis of PMF data obtained from four protein spots revealed the identification of putative transferases. Among them, three (spots 8, 12 and 23) were identified as hypothetical proteins but contained transferase domains. PMFs of spot 19 were resembled with adenylylsulfate kinase or adenosine 5'-phosphosulfate kinase (ASK) that is required for sulfate assimilation and involved in methionine metabolism in yeast [15].

The PMFs obtained from 4 spots (15, 22, 28 and 31) were matched to proteins involved in regulating RNA synthesis. Defective RNA synthesis can impair protein synthesis and other RNA functions. Spot 15 was identified as *M. oryzae *hypothetical protein MG08592.4 that showed 46% similarity to the survival motor neuron protein, Smn1 from *Schizosaccharomyces pombe*. The Smn1 is implicated in RNA splicing. Spot 28 was a hypothetical protein (MGG_03116.5) that showed 68% positive homology with the *Talaromyces stipitatus *cell cycle control protein, Cwf8. Protein spot 31 was similar to a palmitoyltransferase that contained a DHHC zinc finger domain and two putative transmembrane domains (predicted by TMHMM server version 2). Spot 22 were identified as a hypothetical protein and contained domains implicated in RNA synthesis.

The PMFs from three protein spots were matched to proteins involved in nuclear trafficking (spot 17), protein folding (spot 18) and chromosome maintenance (spot 30). Protein spots 17, 18 and 30 were identified as Ran GTPase binding protein Mog1, nucleotide exchange factor Fes1 for heat shock protein 70 (HSP70), and minichromosome maintenance 3 (MCM3) protein, respectively.

In addition, sequence analyses using SignalP algorithm have predicted that three of identified proteins contained canonical hydrophobic signal peptides at their N-terminal regions for secretion. Among them, two (spots 26 and 27) were identified as secreted oxidoreductases and hence grouped under corresponding category. The rest one (spot 14) was identified as a hypothetical protein.

### Identification of conserved domains

Ten (spots 3, 8, 11, 12, 13, 14, 20, 22, 23 and 24) out of 31 differentially regulated proteins were identified as hypothetical, conserved hypothetical and predicted proteins that show no homology to functionally characterized proteins with known functions. These proteins from unknown function were predicted from automated whole-genome sequencing and annotation projects. Further annotation was performed by searching the conserved domains within their amino acid sequences (Table 2). No domain had been detected for 5 proteins (spots 11, 13, 14, 20 and 24). Spot 3 (MGG_02610.5) carries an esterase/lipase conserved domain (Aes, COG0657). Lipases are involved in lipid metabolism. The conserved domain search reveals that spot 8 (predicted protein MGG_05861.5) contains an acetyltransferase domain (RimI, COG0456). This enzyme catalyzes the generalized reaction: acetyl-carrier + reactant = acetyl-reactant + carrier. Acetylation is an important and versatile covalent modification of proteins [16]. Protein spot 11 was resembled with a hypothetical protein MGG_02992.5 with 41% sequence coverage. No domain has been detected in amino acid sequence of this protein. Mutants harboring a T-DNA insertion in the *MGG_02992.5 *gene showed that this gene is indispensable for conidial morphology and conidiation [17]. Hypothetical proteins MGG_09874.5 (spot 12) and MGG_10684.5 (spot 23) carry the conserved domain pfam08241 for S-adenosyl methionine (SAM) dependent methyltransferase and pfam10294 for putative methyltransferase, respectively. Methylation is involved in both gene repression and activation of heterochromatic and euchromatic regions of eukaryotic chromosomes [18]. Protein spot 22 (MGG_10674.5) carries cd04328 that belongs to RPA43, N-terminal ribonucleoprotein domain. RPA43 is a subunit of eukaryotic RNA polymerase I.

**Table 2:** Conserved domains identified in hypothetical proteins.

Spot no.	Conserved domain	CD length	Score (bits)	E-value
3	COG0657, Aes, Esterase/Lipase	312	127.7	3e-30
8	COG0456,RimI, Acetyltransferase	177	50.7	4e-07
12	pfam08241, Methyltransferase	95	35.6	0.007
22	cd04328, N-ribonucleoprotein (RNP) domain	89	90.2	6e-19
23	pfam10294, Putative methyltransferase	172	54.25	4e-08

## Discussion

Over the past decade, *M. Oryzae *has been emerged as a seminal model to elucidate fungal development, infection-related morphogenesis, plant-pathogen interactions and more recently effector biology. A previous report had demonstrated that the *COM1 *gene was indispensable for maintaining normal conidial morphology and full virulence *in M. oryzae*. The *COM1 *orthologs are typically well distributed in filamentous ascomycetes [9] and basidiomycetes. Therefore, we speculate that the aberrant phenotypes attributed to *M. Oryzae *are general features of the *COM1 *among spore-producing ascomycetes and basidiomycetes. In the current study, we exploited proteomics approach to investigate mechanisms underlying conidial morphology regulation and fungal virulence. To the best of our knowledge, there has been no previous proteomics-based analysis on conidial morphology for any fungal pathogen. Moreover, we believe that this is the first attempt to catalogue cellular and molecular components modulating the conidial morphology.

Many phytopathogenic fungi, including *M. oryzae*, synthesize melanin, as it is indispensable for appressorial maturation and the ability to build up the turgor pressure required for mechanical breaching of the leaf surface and thus infecting the host [19,20]. In addition to role in pathogenicity, fungal melanin may also contribute to spore development. Targeted disruption of a gene *BRM2 *encoding trihydroxynaphthalene reductase (3HNR) from *Alternaria alternata *had shown that this gene was required for conidial development. Mutants harboring disrupted *BMR2 *locus exhibited altered conidial size and septal number [21]. In *M. oryzae*, melanin biosynthesis proceeds through a pentaketide route, joining acetate units to make tetrahydroxynaphthalene (4HN) [22,23]. The 4HN is then transformed into dihydroxynaphthalene (2HN) through a succession of two reduction and two dehydration steps. The 2HN is considered to be the ultimate precursor of fungal melanin, a polymer that is exploited by the pathogen during the initiation of disease [19,24]. Both dehydration reactions (scytalone to trihydroxynaphthalene [3HN] and vermelone to 2HN) in the biosynthetic pathway are catalyzed by SCD [25-27], an enzyme that is also the biochemical target of commercial fungicides [28-30], and reduction steps are catalyzed by naphthalene reductases. The 4HNR catalyzes the reduction of 4HN to scytalone and 3HNR converts 3HN to vermelone, an intermediate step in melanin biosynthesis (Figure 7A). The transcriptional regulator COM1, therefore, affects the normal functioning of melanin biosynthetic pathway by modulating the abundance of SCD and 4HNR. The transcriptional downregulation of genes encoding SCD and 4HNR were also observed in the Δ*com1 *mutant, which corroborated the proteomics results (Figure 7B, C &7D). Rather than temporally restricted to the conidia, the altered abundance of SCD and 4HNR may also affect the appressorial development stage, hence directly affect the deposition of melanin between cell wall and plasma membrane of appressoria produced by the Δ*com1 *mutant (Figure 7E). Therefore, partial melanization of Δ*com1 *appressoria may be attributed by the down-regulation of SCD and 4HNR in conidia. Melanization provides physical rigidity to the appressorial cell wall, which is necessary for maintaining turgor pressure and focusing it for vertical penetration [19,20]. Reduced melanization in the Δ*com1 *mutant resulted in less rigid and relatively small sized appressoria than that of P131 strain (Figure 7E). Cytorrhysis assays with mature appressoria indicated that the Δ*com1 *mutant was defective in generating normal appressorial turgor [9]. This turgor defect was due, in part, to the melanin layer of mature appressoria, which had reduced accumulation of melanin. Melanin layer also plays an important role, at least in part, in preventing the leakage of glycerol (main source of turgor generation) and small lipophilic molecules from the cell [31,32]. Melanization defect in the Δ*com1 *appressoria was responsible for suppressed appressorial penetration, which eventually resulted in reduced epidermal cell colonization by invasive biotrophic hyphae. Therefore, attenuated virulence of the Δ*com1 *mutant might be associated with down-regulation of SCD and 4HNR. In addition, mycelia grown in Petri dishes containing complete medium under nitrogen starvation condition showed reduced pigmentation (Figure 7F), which appeared to result from the down-regulation of SCD and 4HNR. The *COM1 *was expressed constitutively in *M. oryzae *in all *in vitro *cell types and *in plant *developmental stages of the pathogen [9]. Therefore, we speculate that the COM1p also regulates melanin biosynthesis during vegetative growth.

**Figure 8:**
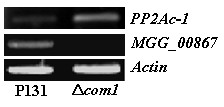
**RT-PCR analysis of genes encoding hypothetical protein MGG_00867.5 and catalytic subunit of serine and threonine protein phosphatase (PP2Ac-1)**. Actin is used as an external standard.

The fungal cytoskeleton is made up of two main structural components: microfilaments (F-actin), which are polymer of the protein actin and microtubules, which are polymer of the protein tubulin. Both microfilaments and microtubules pervade the cytoplasm in what can be quite often intricate three dimensional arrangements, yet they are not simply frameworks around which cells are built or organized. They are in fact very dynamic and play a multitude of roles in fungal biology [33]. The cytoskeleton is important for cell shape in all eukaryotes. Actin related proteins 2 and 3 form major subunits of the ARP2/3 complex, which is known as a key regulator of actin organization in diverse organisms [34-36]. Functional dissection of this complex in *S. cerevisiae *has delineated that ARP2 and ARP3 come together through the concerted movement to form a nucleation site for new actin filament formation. In filamentous fungi, actin filaments are organized as cortical patches that localize to actively growing or emerging hyphal tips and at sites of septation [37]. The loss of either of the two major subunits (ARP2 or ARP3) severely compromises the actin-nucleating activity of the complex. Disruption of ARP2/3 complex subunits in *S. cerevisiae *had resulted in a slow-growth phenotype characterized by defects in cortical actin patches [38]. The COM1 may probably function upstream of the *ARP2 *and regulates its activity. Therefore, deletion of *COM1 *leads to down-regulation of *ARP2 *expression in conidia (Figure 8). The ARP2 might have a crucial role in determining the conidial morphology. However, deletion of the *ARP2 *in *M. oryzae *was lethal, which hampered to investigate the role of *ARP2 *in conidial development. Another structural component of cytoskeleton, microtubules mainly consist of two globular protein subunits, alpha- and beta-tubulins implicated in a wide array of functions, including cell motility and cytoplasmic streaming, nucleus and chromosome movement, maintenance of cell shape, intracellular and axoplasmic transport, and anchorage of cell surface receptors [39]. Therefore, aberrant shape of the conidia produced by the Δ*com1 *mutant might be associated with defect in cytoskeleton organization.

Fungal conidia are equipped with storage compounds like carbohydrates, lipids and proteins. Thus the proper utilization of these compounds is necessary for the fitness or vigor of conidia. The GAPDH plays an important role in glycolysis and gluconeogenesis pathways by reversible catalyzation of the oxidative phosphorylation of D-glyceraldehyde-3-phosphate into 1, 3-diphosphoglycerate [40]. The role of GAPDH in the glycolytic cycle is well known. The GAPDH activity may influence many other cellular activities, facilitating a greater capacity for adaptation to environmental challenges or growth conditions. Moreover, recent studies have shown the importance of certain housekeeping enzymes, including the GADPH as virulence factors for a vast variety of microbial pathogens [41,42]. The m-MDH catalyzes the pyridine nucleotide-dependent interconversion of malate and oxaloacetate in the tricarboxylic acid (TCA) cycle. It is a ubiquitous key regulatory enzyme of the energetic metabolism *via *the TCA cycle, regulating the exchange of metabolites and reducing equivalents across the mitochondrial membrane and in general, the cellular redox state. Oxaloacetate, the product of the reaction catalyzed by MDH, is an oxalic acid precursor [43,44], which has been described as a pathogenicity factor in *Botrytis cinerea *[45]. Both dehydrogenase enzymes are responsible for producing reducing equivalents (NADH) during cellular respiration, suggesting that down-regulation of these enzymes plays a role in reducing energy turnover in conidia produced by the Δ*com1 *mutant, which contributed, in part, to abnormal shape of the *com1 *conidia. In aerobic tissues most ATP is synthesized via the m-ATPase. ATP synthase catalyzes the synthesis of ATP from ADP and Pi. The m-ATPase (F_0_F_1 _ATP synthase) is composed of two units termed F_0 _and F1. F1 has 5 subunits- α, β (spot 10), γ, δ, and ε [46]. The ATP25 protein required for functional F_0 _was found to be down-regulated in the conidial proteome of Δ*com1 *mutant. Mutation in *ATP25 *prevented the assembly of functional F_0 _unit of the m-ATPase in *S. cerevisiae *[47]. In contrast to the m-ATPase that exploits a proton gradient to generate ATP, the v-ATPase utilizes the energy arising from ATP hydrolysis to actively transport protons into organelles like vacuoles and extracellular compartments, thereby acidifying organelles. The acidification of fungal vacuoles is crucial for protein turnover, cellular homeostasis, membrane trafficking, signaling and nutrition. These functions are vital for fungal growth, differentiation and pathogenesis [48]. The v-ATPases are multi-subunit complexes composed of two functional domains (V_1 _and V_0_). The peripheral V_1 _domain complex responsible for ATP hydrolysis contains at least eight different subunits (subunits A-H). The H-subunit (spot 29) plays an essential regulatory role. Therefore, we speculate that the aberrant morphology and reduced virulence of Δ*com1 *mutant might, in part, be associated with reduced energy level and defective cellular homeostasis.

Another category of proteins regulated by COM1p was oxidoreductases or related proteins. GcdH is a unique member of the acyl-CoA dehydrogenase family of flavoproteins owing to its additional catalytic function of decarboxylation [49]. In eukaryotes, GcdH is a key mitochondrial enzyme involved in the catabolic pathways of the amino acids, such as L-tryptophan, L-lysine and L-hydroxylysine. It catalyzes both steps (dehydrogenation and decarboxylation) of the conversion of glutaryl-CoA and FAD to crotonyl-CoA, CO_2 _and FADH2 [50]. Keon et al. [51] identified GcdH from EST library of the wheat fungal pathogen *Mycosphaerella graminicola *utilizing ammonium as a sole nitrogen source. Another acyl-CoA dehydrogenase identified in the conidial proteome of Δ*com1 *mutant was IVD. IVD is a secreted oxidoreductase, which catalyzes the third step of leucine catabolism: conversion of isovaleryl-CoA (3-methylbutyryl-CoA) into 3-methylcrotonyl-CoA [52]. Many filamentous fungi like *A. nidulans *are able to utilize amino acids as sole carbon source. These fungi catalyze leucine to acetyl-CoA and acetoacetate. This acetyl-CoA can then be converted into carbohydrates using the glyoxylate bypass of the TCA cycle [53]. A molybdopterin binding domain protein was identified as COM1-induced protein. Eukaryotic and prokaryotic molybdoenzymes require a molybdopterin cofactor for their activity. Molybdopterin cofactor renders the catalytic activity to these enzymes. Many eukaryotic oxidoreductases like xanthine dehydrogenase, aldehyde oxidase, nitrate reductase and sulfite oxidase require and bind a molybdopterin cofactor for their activity [54]. These enzymes are important to maintain cellular redox homeostasis. Up-regulation of molybdopterin binding domain protein could lead to disturbance in the cellular redox, which may result in morphological and physiological defects. Filamentous fungi use several catabolic pathways to derive energy and structural components from organic compounds like phenylacetate and amino acids. For instance, *A. nidulans *utilizes phenylacetate as a carbon source via homogentisic acid pathway. Phenylacetate 2-hydroxylase catalyzes the first step of phenylacetate catabolism, which ultimately leads to the production of acetoacetate and fumarate [55]. The fumarate can feed into the TCA cycle and optimize cellular energy production. Our study has shown that the COM1p modulates the abundance of oxidoreductases, thereby also affects the energy turnover from alternative pathways.

Deletion of the *COM1 *also modulated the abundance of two hydrolases, viz., PP2Ac and a lipase domain containing protein. Protein phosphorylation is a reversible mechanism, catalyzed by protein kinases and protein phosphatases (PP), which is thought to be one of the important regulatory processes in eukaryotic cells. As protein phosphatases are an integral part of the reversible phosphorylation regulatory machinery, they are key elements in maintaining the balance of many cellular activities. On the basis of substrate specificity, they are classified as serine/threonine, tyrosine, dual specificity, or histidine protein phosphatases. Former classification has grouped serine/threonine protein phosphatases as PP1 and PP2 (A, B, C) [56]. PP2A, a member of the serine/threonine protein phosphatases subfamily, plays an essential role in the regulation of a wide range of metabolic and cellular processes, including cell motility, cell division, growth, signaling and gene expression [57,58]. The PP2A holoenzyme consists of a core complex comprising a 36 KDa catalytic subunit (PP2Ac) tightly associated with a 65 KDa regulatory subunit (A). *In silico *analysis has revealed that *M. oryzae *strain 70-15 possesses three genes encoding PP2Ac: *MGG_06099.6 *(*PP2Ac-1*), *MGG_01690.6 *(*PP2Ac-2*), and *MGG_01528.6 *(*PP2Ac-3*). Sequence alignment analysis showed that polypeptides encoded by these genes share >70% positive homology with each other (Additional file 4). The PP2Ac-1 (spot 25) showed induction in its expression level upon *COM1 *deletion (Figure 4 and Figure 5). RT-PCR also corroborated the induction of gene encoding PP2Ac-1 at transcriptome level (Figure 8). It might be possible that the COM1p functions upstream of the PP2Ac-1. Targeted deletion of genes encoding PP2Ac-1 and PP2Ac-2 revealed that PP2Ac-1 is indispensable for conidiation and fungal growth, whereas PP2Ac-2 is required for asexual development and pathogenicity of *M. oryzae *(unpublished data). In *Neurospora crassa*, inactivation of *pph-1 *(ortholog of *pp2ac-1*) was lethal. Nuclei harboring a disrupted *pph-1 *gene could only be maintained in a heterokaryon, indicating that the expression of *pph-1 *is essential for fungal growth [59]. Therefore, the essential function of PP2Ac-1 might be specific feature of this species and related species. Lipase catalyzes the hydrolysis of lipids into glycerol and fatty acids. Lipid bodies, glycogen and disaccharide trehalose are the most abundant carbon storage products in conidia and among them, lipids contribute to glycerol production in appressoria [4,31]. Transmission electron micrographs of conidia revealed that Δ*com1 *conidia had significantly reduced lipid body like droplets (LBDs) than those of the strain P131. Up-regulation of lipase activity could lead to high glycerol level, which, in turn, can increase turgor generation in the Δ*com1 *appressoria. However, cytorrhysis assays delineated that the Δ*com1 *appressoria were defective in turgor generation mainly because of reduction in LBDs in conidia [9]. Therefore, it might be possible that a part of glycerol produced by lipolysis of lipids is leaked out from the Δ*com1 *appressoria because of reduced melanization. This further reduces appressorial turgor pressure, which eventually leads to a penetration defect.

Transferase activity of several enzymes was also influenced by COM1p in the conidial proteome. Interestingly, a transferase kinase ASK is only assimilated in the Δ*com1 *mutant conidia. In yeast, ASK is required for sulfate accumulation and implicated in methionine metabolism [15]. Sulfur assimilation is indispensable to maintain the yeast phase of the dimorphic fungus *Paracoccidioides brasiliensis *[60,61]. Two methyltransferases (spots 12 and 23) were found to be induced in the Δ*com1 *mutant conidia. One of them was SAM-dependent methyltransferase involved in the modification of a large number of substrates, including proteins, RNA, DNA, and lipids [62,63]. Another differentially regulated transferase was acetyltransferase required for acetylation (an important and versatile covalent post-translational modification of proteins) [16]. We also identified a palmitoyltransferase Pfa4. Palmitoylation, which is the thioester linkage of palmitate to selected cysteine residues, is one of several lipid modifications used for tethering proteins to membranes [64]. In yeast, Pfa4 is required for chitin synthase 3 (Chs3) palmitoylation, which is indispensable for ER-export of Chs3. Lam et al. [65] suggested a more generalized role of palmitoylation in the ER quality control of polytopic membrane proteins. The abundance of pfa4 was abolished in the Δ*com1 *conidia, delineating defective ER-export of certain group of proteins, including chitin synthase.

Our data suggest that three proteins (Smn1, Cwf8 and RPA43) involved in RNA synthesis were also affected by the *COM1 *deletion. Among them, two Smn1 and Cwf8 were only detected in the Δ*com1 *conidia, whereas RPA43 was undetectable. Smn1 protein is involved in small nuclear ribonucleoprotein biogenesis for mRNA splicing [66]. Cwf8 protein is the apparent ortholog of the *S. cerevisiae *splicing factor Prp19. The Yeast Prp19 is involved in mRNA processing and splicing, and ubiquitin conjugation pathway [67]. RPA43 is a subunit of eukaryotic RNA polymerase I. In yeast, inactivation of gene encoding RPA43 leads to a lethal phenotype. This lethal phenotype could be limited to defective spore germination [68]. RPA43 homologues in the Δ*com1 *mutant may remain their activity as they are important for vitality.

A protein Mog1 involved in nuclear trafficking was also abrogated in the Δ*com1 *conidia (Figures 4, 6 &8). Oki and colleagues [69] originally identified Mog1 protein during screening for multi-copy suppressors of temperature-sensitive alleles of the *S. cerevisiae *Ran homologue, Gsp1p, which were defective in nuclear trafficking. The Mog1 protein binds to the small GTPase Ran that plays an important role in nuclear import. Binding is independent of Ran's nucleotide state (RanGTP/RanGDP). The transport of proteins and nucleic acids through nuclear pore complexes relies on a series of coordinated interactions involving both soluble transport factors and nuclear pore complex components [70-72]. Therefore, it can be predicted that the COM1p is required to turn on the expression of *Mog1*. We also identified a nucleotide exchange factor FES1 for HSP70 molecular chaperone, which facilitates protein synthesis, folding, transport, and degradation. The FES1 catalyzes the release of ADP from Hsp70, allowing the binding of ATP, and thereby drives the protein folding cycle forward [73-76]. A COM1p-induced MCM3 protein was also extracted from the Δ*com1 *conidia. The MCM proteins are essential for the initiation of DNA replication in eukaryotes [77]. Deletion of the *Sordaria macrospora *gene *mcm1*, which encodes a *S. cerevisiae *MADS box protein resulted in reduced biomass, increased hyphal branching and reduced hyphal compartment length during vegetative growth [78].

## Conclusions

In summary, we catalogued cellular and molecular components regulated by the conidial morphology modulating gene *COM1*. The current study will be instrumental in deciphering the mechanisms underpinning conidial development, normal conidial morphology, and plant infection in conidia producing phytopathogenic fungi.

## Methods

### Fungal strains, growth conditions and collection of conidia

The P131 [79] and Δ*com1 *strains used in this study were the *M. oryzae *wild-type field isolate and *COM1 *gene deletion mutant in the P131 background [9], respectively. Fungal strains were routinely maintained in Petri dishes (Ø 9 cm) containing the oatmeal tomato agar (OTA) medium (150 ml tomato juice/liter, 40grams oat/liter, 14-16 grams agar/liter) at 25°C.

For harvesting conidia, the conidial suspension was spread and incubated on OTA plates at 28°C for 48 hours. The aerial mycelia were then removed with a sterilized loop and incubated at 28°C under fluorescent light to induce synchronous conidiation. After 48 h of incubation, the conidia were placed into double distilled water containing 0.02% v/v tween-20 and filtered through the four layered Kimwipe towel. The conidia were pelleted by centrifugation at 1800 × g for 10 min and washed twice with double distilled water. The pellet was then frozen in liquid nitrogen and stored at -80°C until required.

### Protein extraction

Total proteins were extracted from one gram each of the strain P131 and Δ*com1 *conidia using Mg/CHAPS extraction buffer [80]. The conidia were ground into fine powder in liquid nitrogen with a pre-cooled mortar and pestle, and homogenized in 5 ml of ice cold Mg/CHAPS extraction buffer containing 0.5 M Tris-HCl (pH 8.3), 2% CHAPS, 20 mM MgCl_2_, 2% w/v DTT and 1 mM PMSF. After centrifugation at 12000 × g for 15 min at 4°C, the supernatant was precipitated with 4 volumes of ice chilled 10% TCA in acetone with 0.007% DTT. Proteins were pelleted by centrifugation at 3000 × g for 10 min (4°C) and the pellet was washed thrice with acetone containing 0.007% DTT. Subsequently, the pellet was thoroughly resuspended in rehydration buffer [7 M urea, 2 M thiourea, 4% CHAPS, 2% immobilized pH gradient (IPG) buffer (pH 3-10 L or pH 4-7 L) and 20 mM DTT]. Protein concentration was estimated using the Bradford assay with BSA as a standard [81].

### Cataloguing proteins by 2-DE

The 2-DE was performed with Ettan IPGphor system (Amersham Biosciences, Sweden) for first dimension isoelectric focusing (IEF). The IPG strips (pH 3-10 Linear, 18 cm and pH 4-7 Linear, 18 cm) were rehydrated overnight in the re-swelling tray with 340 ul of rehydration buffer containing 200 ug of total proteins. Focusing was performed through 1 h at 300 Volts (step and hold mode), 2 h at 500 Volts (step and hold mode), 6 h at 1000 Volts (gradient mode), 3 h at 8000 Volts (gradient mode) and 1 h 10 min at 8000 Volts (step and hold mode). After IEF, the strips were equilibrated twice with gentle shaking for 15 min in the sodium dodecyl sulfate (SDS) equilibration buffer. The first step was performed in an equilibration solution containing 50 mM Tris-HCl (pH 8.8), 6 M urea, 30% v/v glycerol, 2% w/v SDS, 0.002% w/v bromophenol blue, and 1% w/v DTT. The second step was performed in an equilibration solution in which the DTT was substituted by 2.5% w/v iodoacetamide. The second dimension separations (on 12.5% polyacrylamide SDS gels) were carried out with Ettan Dalt Six (Amersham Biosciences, Sweden). The 2-DE gels were then silver stained. Four independent biological replicates per IPG strip were used for each strain to compare the conidial proteomes.

### Image scanning and analysis

The silver-stained gels were scanned at 600 dots per inch (dpi) resolution using Image Scanner (Amersham Biosciences, Sweden). The transparency mode was used to obtain a grayscale image. The image analysis was performed with ImageMaster 2D Platinum software version 5.0. The optimized parameters were as follows: saliency 2.5 and minimum area 50.

### In-gel digestion

The protein spots were excised into 1 mm pieces and placed into low-binding Eppendorf tubes. The spots were then washed with deionized water for 10 minutes with intermittent vortexing and destained twice for 15 minutes with intermittent vortexing with 200 ul of freshly prepared 1:1 solution of 100 mM sodium thiosulfate and 30 mM potassium ferricyanide. Supernatants were discarded. Slices were washed with ultra pure water and equilibrated for 10 minutes with intermittent vortexing in 200 ul of 25 mM ammonium bisulfate. These spots were then dehydrated twice for 10 minutes vortexing with 200 ul of 25 mM ammonium bicarbonate/50% acetonitrile (ACN). Supernatants were poured off. After drying in a speed vacuum centrifuge for 10 minutes, the gel slices were rehydrated for 15 minutes at 4°C in 25 ul trypsin (Sigma, Proteomics Grade) in 25 mM ammonium bicarbonate (pH 8-8.5) and overlaid with minimum volume of 25 mM ammonium bicarbonate to keep them immersed throughout the digestion process and incubated for 16-24 h at 37°C. The digest solution was transferred to a new low binding micro-centrifuge tube. The digested peptides were extracted with one volume (40-50 ul) of 0.1% trifluoroacetic acid (TFA) in 50% ACN by vortexing 20 minutes at room temperature. This solution was combined with digest solution.

### MALDI-TOF MS analysis

The resulting peptide mixtures were desalted using Zip-Tip C18 (Millipore) and eluted onto the stainless steel MALDI target. Two microliters of the sample on the target plate were mixed with 1 ul of 10 mg/ml a-cyano 4-hydroxy cinnamic acid in 0.1% TFA. The **s**amples were then allowed to dry at room temperature. The MS analysis was conducted by MALDI-TOF/TOF mass spectrometer (Auto Flex II, Bruker Daltonics) in the positive ion reflector mode. Calibration was carried out using a standard peptide mixture. The mass spectrometric data (PMFs) were collected from monoisotopic peaks falling in the m/z range of 750-4000 Da.

### Database search

The PMF data obtained from MS were utilized for protein identification by analyzing the sizes of tryptic fragments via the MASCOT (Matrix Science) search engine using the entire NCBI non redundant fungal protein database. For effective PMF analysis, it was assumed that peptides were monoisotopic and the possibility that methionine residues oxidized was considered. The fingerprinting method allowed for a maximum of one missed tryptic cleavage per protein. The maximum deviation permitted for matching the peptide mass values was 100 ppm.

### Identification of conserved domains

To determine the putative functions of hypothetical, conserved hypothetical and predicted proteins from *M*. oryzae, we used the obtained sequence information to search for the conserved domains (CDs) using an on-line CD-Search software available at http://www.ncbi.nlm.nih.gov/Structure/cdd/wrpsb.cgi The newly developed CD database was searched against for all possible CDs [82]. The CD with the highest score was listed as the CD for the respective hypothetical, conserved or predicted protein.

### RNA extraction and semi-quantitative RT-PCR

Total RNA from 100 mg frozen conidia of the strain P131 and the Δ*com1 *mutant was isolated using Redzol reagent (SBS Genetech, Beijing) following the protocol of the supplier. The genomic DNA was eliminated using RNase-free DNaseI (Takara) from isolated total RNA and stored frozen at -80°C until required. Reverse transcription was performed in a total volume of 20 ul using 10 Units AMV RTase (Takara), 10 mM of dNTP mixture (Takara), 50 pmol poly (dT)_18 _primer (Takara) in reaction buffer with 120 ng of the DNA free conidial total RNA. The reaction mixture was incubated for 60 minutes at 42°C, followed by 15 min at 70°C for inactivation of reverse transcriptase. The resultant cDNAs were subjected to PCR amplification. PCR amplification was performed using 28 cycles. The PCR products were separated on 1% agarose gel and stained with ethidium bromide. RT-PCR was performed as duplex RT-PCR using actin as a standard in a Primus Thermocycler (MWG-Biotech, England). Primers used in the RT-PCR analysis are listed in table 3.

**Table 3:** Primers used in this study (F, Forward; R, Reverse)

Genes	Primers
*SCD*	F: 5'-TGACTACCGCTCCTTCCT-3'
*SCD*	R: 5'-CCGTCCGTCCTCAAAGAT-3'
*T4NR*	F: 5'-GTCCTCAGTCAGCCTCTCTT-3'
*T4NR*	R: 5'-CACGGGTGTTGATGGTAAAG-3'
*MGG_00867.5*	F: 5'-CGCACTACAAGAACACAAG-3'
*MGG_00867.5*	R: 5'-TAGACCTCGTTGAACAGC-3'
*PP2Ac-1*	F: 5'-GGATGGATGGATTGAGAG-3'
*PP2Ac-1*	R: 5'-TGTAAGAGGCAGGTAGTC-3
*Actin Actin*	F: 5'-TGGCACCACACCTTCTACAA-3'
	R: 5'-CGGAGTCGA-GCACGATACCA-3'

## Competing interests

The authors declare that they have no competing interests.

## Authors' contributions

YLP designed the research. VB conducted the experiments and wrote the manuscript. LXW participated in the mass spectrometric data analyses. All authors have read and approved the final manuscript.

## Reviewers' comments

### First round

#### Reviewer's report 1

Dr. George V. Shpakovski, Shemyakin-Ovchinnikov Institute of Bioorganic Chemistry, Russian Academy of Sciences, Moscow, Russian Federation

The paper describes the mass spectrometry (MALDI-TOF) based proteomics study of the fungal phytopathogen *Magnaporthe oryzae *that causes rice blast, one of the most devastating diseases of *Oryza sativa *L. species worldwide. Because the conidia produced by this fungal pathogen are the main source of disease dissemination and the morphology of conidia may be a decisive factor in the initiation of plant infection and spreading of blast disease, the authors have compared the conidial proteomes of *M. oryzae *wild-type strain P131 and Δ*com1 *mutant defected in the *COM1 *(***Co****nidial ****M****orphology ****1***) gene. In total, the production of 31 proteins was found to be significantly different in the two strains.

The work is carefully done, thoroughly described and certainly deserved to be published. It is probably the first attempt to catalogue cellular and molecular components modulating conidial morphology of a fungal pathogen.

Concerning the results obtained, I am a bit surprised that two conidial proteomes comparisons shown in the paper (on pH scales 3-10 and 4-7) gave completely different sets of proteins (eleven proteins in the first case and twenty in the second). This fact needs to be explained. The authors claimed that their results are highly reproducible, but they did not mentioned in the paper's text how many independent experiments were actually carried out.

The authors claim that "RPA43 was not expressed ... in the Δ*com1 *conidia" looks also quite dubious or at least 'too categorical' for me... Indeed, *RPA43 *is a vitally important gene in *S. cerevisiae*: its product, the RNA polymerase I subunit RPA43, provides an important interface for communication of the rRNA synthesizing machinery with the transcription factor RRN3. Even if the level of rRNAs is substantially reduced in the Δ*com1 *conidia, the *M. oryzae RPA43 *homologue must be expressed at a somewhat detectable level.

I also have one more general comment. The paper is nicely written, but the authors need to be aware of that strictly speaking the production (the synthesis) of a protein is the result of the expression of a genetic determinant (gene or cDNA), and proteins 'express' themselves through their plentiful activities such as catalytic action, DNA binding, etc. None of the protein activities were actually measured in this work, so it is better to avoid the phrases of a type "the protein was not expressed", "the expression levels of proteins" throughout the paper' text.

There is also a spelling error on the page 5 of the manuscript: it must be "Archiascomycetes" instead of "Archioascomycetes"...

#### Authors' response

*Four independent biological replicates per IPG strip were used for each strain to catalogue the conidial proteomes of M. oryzae wild-type strain P131 and Δcom1 mutant. We mentioned it in revised version. As mentioned above, we repeated our experiments 4 times and all the time we got similar spot pattern. Therefore, we concluded that the pattern was real and caused by expanding the pI (4-7) on same length of IPG strip as 3-10 pI*.

*I am completely agreed with the reviewer on RPA43 that description is too categorical. Indeed we can not rule out the possibility of expression of RPA43 homologues. Therefore, we tried to avoid categorical statement in the text*.

*We omitted the phrase like "the expression of proteins" in the text and deleted Archiascomycetes in the final version*.

#### Reviewer's report 2

Dr. Karthi Sivaraman, EMBL Outstation - Hinxton, European Bioinformatics Institute, Wellcome Trust Genome Campus, Cambridge, United Kingdom

##### Overview and importance of the work

*Magnaporthe oryzae *or the rice blast fungus is the leading cause of fall in rice productivity worldwide. Previous work has revealed the importance of fungal conidia formation in the pathogenesis process, and a role for Com1 protein in regulating conidia formation. In this work, the authors have studied the effect of com1 deletion on the proteome of *M. oryzae*. They find 31 proteins that are differentially present in the proteomes of wild type and com1 deletion mutant of *M. oryzae*. This study has both biological and economic importance due to its relevance to agriculture and food security.

##### Review Summary

The paper is an important work that bridges our knowledge gap in *Magnaporthe oryzae *biology. The authors should address all the major concerns raised. The minor concerns can be dealt with, at the editor's discretion. Given the biological and economic importance, I recommend the paper for publication after the major concerns are addressed.

##### Major concerns

There are several important concerns that need to be addressed.

1. Are the strains (P131 and Δcom1) isogenic? If so, the authors should state it explicitly. If not, how do the authors control for strain specific variations?

2. Why did the authors use two pI ranges in the 2D-electrophoresis experiments?

3. Why are the results so different? Why did not the authors pick up the second set of proteins (from the pH4-7 gel) from the first gel itself (pH range 3-10)?

4. The authors mention that the experiment was repeated 4 times. Was it repeated 4 times for each strain? Or was it twice per strain? What was their overall reproducibility - for example, how many spots were reproducibly found in each repetition? What is the probability of missing a spot? The authors need to provide statistics for the same.

5. How do the authors define "differentially regulated"? What was the statistical test? What was their p-value cutoff?

6. Figure 6 shows all numbers in percentages. Since there are only 30 genes, showing them in percentages could be misleading to a reader. For example, ~7% of 30 is only 2 in absolute numbers. The graph should be reworked to show absolute numbers.

7. The authors have used COG for functional classification of proteins. Since eukaryotes have a lot of functional information that cannot be essentially captured by COG classification, the authors should consider using GO classification instead.

8. "Identification of conserved domains" is a part of functional characterization and should be treated as such.

9. The Methods section does not provide sufficient information about the statistics that were used in the paper. The authors have to rigorously satisfy statistical significance using a) t-test for significance of spot differences/semi-quantitative RT PCR, or b) Fisher's exact test for categorical data (for functional enrichment), as is the case.

10. In the methods, the authors should describe how they chose regions on the gel that they eventually analyzed. They have made a passing reference to this in the results section. This should be detailed in the methods instead.

##### Minor concerns

1. The first part of the results describing the 2D page is not clear. It can be rewritten succinctly.

2. In many cases, the authors introduce snippets of methods in the results section.

3. The authors refer to the Figure1 in their introduction. If the figures were generated by their own work for this paper and for the first time, the authors should add that to the results section. If not, and if the figures (or similar pictures) have been published, the authors should refer to any prior publication.

4. In the table, please provide a column specifying the P-value of differential presence.

5. In figure 7F, add the scale information. The "scale bar" is present but the scale information is not.

6. When the authors mention RNA synthesis, do they mean "transcription"? Or do they mean "nucleotide biosynthesis"? Make clear.

#### Authors' response

First I would like apologize to the reviewer for the mistake in sending additional files, which can answer some of major concerns. Rebuttal for each major concern is appended below:

*Strains P131 and Δcom1 used in the study were wild type and COM1 gene deletion mutant of M. oryzae, respectively (as mentioned in abstract, introduction and methods)*.

*To get broader snapshot of differentially regulated proteins, we used two pH scales. We conducted our all experiments 4 times and all the time we got similar spot pattern. Therefore, we concluded that the pattern was real and caused by expanding the pI (4-7) on same length of IPG strip as 3-10 pI. However, it was not possible to pickup pI 4-7 section from pI 3-10 (because of protein accumulation in 4-7 pI range)*.

*Four independent biological replicates per strain were used for each pI scale. Over 90% spots were found to be reproducible. We considered only spots for MS analyses, which showed differential regulation on all 4 gels compared to wild type*.

*We used Image Scanner software to measure per cent intensity of each differentially regulated spots and confirmed the variation by student's t-test (p values are mentioned in the additional file *2*)*.

*Figure *6*was revised and absolute number was added in it. Proteins that possess known domains are the part of functional classification*.

*Differentially regulated proteins were first identified on gels using Image Master Platinum software. The areas where these proteins located were enlarged*.

*Figure *1*was exclusively generated for this study. All P-values were added in additional file with other statistical analyses. Scale for Figure *7F*was added. RNA synthesis was mentioned in the context of transcription*.

#### Reviewer's report 3

Dr. Lakshminarayan M. Iyer, NCBI, NLM, NIH, Computational Biology Branch, Bethesda, U.S.A.

The authors investigate the consequence of deleting the COM-1 protein that regulates conidial formation in Magnaporthe. Using MALDI-TOF MS, they identify 31 genes that show altered expression in Com1 knockout cells. Interestingly the Com1 protein appears to regulate a disparate set of proteins involving various functional types. One such pair of genes identified in this study is involved in melanin biosynthesis which is known to be critical for appressorial maturation. While the roles of a few other proteins can be linked to conidial formation (e.g. conidial structure), most of the remaining genes appear to have a more general role in cellular metabolism. Of particular interest in this study are proteins that are poorly understood in sequence but yet might be important for the infection or conidiation in a lineage specific way. In general, this is a well designed study and the results merit publication.

However, I do have a few additional comments and quibbles.

##### Major comment

The Com1 protein is poorly analyzed. While a "putative bHLH" has been previously recognized in it, there is no support for such a prediction. In fact there is a fairly well conserved domain, whose function once seen would significantly clarify the role of COM1. Isolating this domain is also quite straight-forward. I shall not reveal the domain, but here's a hint- Run region ~150-300 of COM1 through profile- profile or iterative PSI-BLAST searches.

(Since the ELL domain has been discovered in COM1, it needs to be appropriately cited (PMID: 17150956). Also, in my opinion, COM1 is orthologous to S. pombe ELL. Note that the best hits of S. pombe ELL are also the best hits of COM1. The S.pombe protein has diverged and hence does not appear in the first pass of blast searches. Orthologs of ELL are also present in basidiomycetes (e.g. CND01350 from Cryptococcus neoformans)and so it is not right to say that COM1 (an ortholog of ELL) is missing in basidiomycetes. There is no helix-loop-helix domain. Do the authors mean a basic-helix-loop-helix domain (bHLH) or a helix-turn-helix domain. I dont think there is any support for the presence of the bHLH in these proteins. I would recommend that the authors delineate the regions of the ELL domain (and if supported the bHLH) for clarity.)

#### Minor comments

1. In my opinion, the text, in particular the discussion, can be significantly shortened. For example, Table I could have the functional categories as an additional column and proteins can be grouped by their predicted functional roles rather than the sequential spot number. This could significantly cut down the redundancy in the text. In my opinion, not all genes need an explanation. For example the targets of the dehyrogenases are not exactly known, so assigning a role in amino acid dehydrogenation is rather speculative. Similarly, the two methylases described in this study are not DNA methylases. DNA methylases have well characterized sequence signatures not present in these proteins.

2. Spot 21 labelled as MGG_08809.5 has a Saccharomyces ortholog whose role fits in with some of the other proteins observed in this study. Please revise.

3. Figure 6. The numbers in the pie-chart are a bit misleading especially since the sample size is small. For example, 3% would actually represent 1 protein! Perhaps the actual number of proteins may be added in the brackets.

4. Please change the sentence "In higher eukaryotes, the cytoskeleton plays an important role in determining cell shape." The cytoskeleton is important for cell shape in all eukaryotes.

#### Authors' response

*The COM1 contains a putative ELL domain, which further corroborates its function as a transcription regulator. I have delineated it in the background section that COM1p encompasses an ELL domain (PMID: 17150956) and omitted that it possesses helix-loop-helix structure. Description regarding methylases has been deleted in the revised version from the discussion section. MGG_08809.5 (spot 21) has a homolog ATP25 in Saccharomyces. ATP25 is required for assembling functional F_0 _unit of the m-ATPase. We have added number of proteins under each functional category numbers in the Figure *6*(% in parentheses). The sentence "In higher eukaryotes, the cytoskeleton plays an important role in determining cell shape" is replaced by "The cytoskeleton is important for cell shape in all eukaryotes"*.

### Final remarks by reviewers

#### Dr. George V. Shpakovski (Reviewer 1)

Thank you for sending me the revised manuscript. I am agree with your corrections and do not have further remarks.

#### Dr. Karthi Sivaraman (Reviewer 2)

Thanks for sending me the revised manuscript.

You have addressed almost all of my concerns. The answer for the first concern (regarding isogenicity) is unclear. I gather from your answer that the com1 mutant is derived from the wild type you have used in the study. This relation between the two strains is largely implied. It would suffice if you make it explicit, right in the introduction (and hence my insistence on the term "isogenic").

Otherwise, I am satisfied with the author responses and you can proceed with the publication.

#### Authors' response

*I have made it clear in the background section that COM1 mutant is an isogenic strain of M. oryzae wild type isolate P131*.

#### Dr. Lakshminarayan M. Iyer (Reviewer 3)

All my comments have been addressed in the revised manuscript and I recommend its publication.

## Supplementary Material

Additional file 1 **The COM1p sequence contains a putative ELL domain**. Click here for file

Additional file 2 **Per cent intensities and abundance histograms of identified proteins**. Click here for file

Additional file 3 **Details of identified proteins**. Click here for file

Additional file 4 **Alignment of the predicted amino acid sequence of MGG_06099.6 (PP2Ac-1), MGG_01690.6 (PP2Ac-2) and MGG_01528.6 (PP2Ac-3) using ClustalW algorithm**. Click here for file
