# Supplementary material for: Proteomic changes associated with deletion of the Magnaporthe oryzae conidial morphology-regulating gene COM1
Source: Biol Direct. 2010 Nov 2;5:61. doi: 10.1186/1745-6150-5-61 (PMC2989938; doi:10.1186/1745-6150-5-61)
Supplement: Additional file 1 — The COM1p sequence contains a putative ELL domain. [file 1745-6150-5-61-S1.DOCX]

COM1p (*Magnaporthe oryzae*)

1 MSLVIPAGGL ELETSVDKMT SLPLQAFAIT LSDDMLEDLI DSFQNGQEIE LSLGQSPAFL

61 YGGNTAPITR IPENFSYDLY VTDPNSPETA SLAPNPTMPI FKKQHLNLVK KPKYMPKGFI

121 DEDEAPELGA LEIIENPEKS QTSSQSKSSS LQPQSALNKK PKSATAGKKT AASNAITMST

181 QARSRPTSPA ISAVGSPLPE SSIESSHQQI VKEAKELRAP LIHALAVREM TYDELWEKWG

241 KGDDDESRRE FRNILSKVAE QVKNSNKYMM KKNHWKELDV WNHNYDSDSD RQTAIDNAVR

301 HFDKMRIGAS EPEWQKLLPF DDRGKGKCLS KLQASLARGP KGLTVHVQNA DDTSGAGSPD

361 TDNRSISGSQ PMSRSSSQNK PKKAVEKKPA VSAPKKPTAP KVSPSKPAAK PAAKTGARGP

421 LSKEIITDSD ESSDEIPLSQ TKNVVKKQAA PAVRAPKPPI SAPVSGPSSL PKKPPPPAAR

481 EPAKPQITAK PPVKRPREEE DSSSSSGTPL AKKFKVKEPV RAPKEPIRAP KEPIRAPKET

541 TVRAPKEVIR APRETKPTKE PKAAPLPVSK PRPADSSQST SRTGSSNISF NRSKNTSPAK

601 SSPLASSPPT NASDIDPAEE AMIANANRKR KADAYYNDSS STTSSSSSNV QTSGKNSIKK

661 RTHDDDVSVS RRGAGSKLPP DVVAKARRFK EAYSDYERLH YELSGMNNPE ESKLNELMNM

721 HRRLEKMKKE IYSTTGATYN GDRDRAHKSG EQKRSSAVAS SRRERDEYND YGRH

Underlined sequence represents a putative ELL domain (PMID: 17150956).

[seqsig_9690a4516fe69fe4c42f9296f141e64f](http://www.ncbi.nlm.nih.gov/entrez/query.fcgi?cmd=Search&doptcmdl=GenPept&db=Protein&term=seqsig_9690a4516fe69fe4c42f9296f141e64f) 1 TSPAISAVGSPLPESSIESSHQQIVKEA---KELRAPLIHALAVREMTYDELWEKWGK-GDDDESRREFRNILSKVAEQV 82

[Cdd:pfam10390](http://www.ncbi.nlm.nih.gov/entrez/query.fcgi?cmd=Search&doptcmdl=GenPept&db=CDD&term=pfam10390) 166 SSPLASNRKQSLPGNGSSSSRKANGSSAvmkRPLRERVIHLLALKPYKKPELLLRLQKdGLADKDKNSLDSLLQQVANLN 245

[seqsig_9690a4516fe69fe4c42f9296f141e64f](http://www.ncbi.nlm.nih.gov/entrez/query.fcgi?cmd=Search&doptcmdl=GenPept&db=Protein&term=seqsig_9690a4516fe69fe4c42f9296f141e64f) 83 KNSNKYMMKKNHWKELDvWNHNYDSDSDRQ 106

[Cdd:pfam10390](http://www.ncbi.nlm.nih.gov/entrez/query.fcgi?cmd=Search&doptcmdl=GenPept&db=CDD&term=pfam10390) 246 PKDNSYTLKDCVYKEVQ-EDWPGYSEDERQ 274
