# Supplementary material for: Proteomic changes associated with deletion of the Magnaporthe oryzae conidial morphology-regulating gene COM1
Source: Biol Direct. 2010 Nov 2;5:61. doi: 10.1186/1745-6150-5-61 (PMC2989938; doi:10.1186/1745-6150-5-61)
Supplement: Additional file 3 — Details of identified proteins. [file 1745-6150-5-61-S3.DOC]

1. Proteins (Spot 1-11) identified by Matrix Assisted Laser Desorption Ionization- Time of Flight Mass Spectrometry (MALDI-TOF MS). These proteins were differentially expressed on pH scale 3-10.

**Spot 1.**

**m/z ratio**

3245.726, 3039.276, 2501.884, 11788.884, 1544.675, 1316.558, 1016.117, 998.140, 988.107

Match to: **gi|39940362,** **Scytalone dehydratase [Magnaporthe grisea 70-15]**

Score: **80**

Number of m/z values searched: **9**

Number of m/z values matched: **6**

Sequence Coverage: **52%**

Matched peptides shown in **bold**

1 MGSQVQK**SDE ITFSDYLGLM TCVYEWADSY DSK**DWDRLRK **VIAPTLRIDY RSFLDKLWEA**

61  **MPAEEFVGMV SSKQVLGDPT LRTQHFIGGT R**WEK**VSEDEV IGYHQLR**VPH QRYKDTTMKE

121 VTMKGHAHSA NLHWYKKIDG VWKFAGLKPD IRWGEFDFDR IFEDGRETFG DK

Protein Length=172

Theoretical MW=20246.4, pI=6.25

Nominal mass M_r_= 20237, Calculated pI value=5.87

**Spot 2. MGG_10690.5**

**m/z ratio**

2661.975, 2582.936, 2292.543, 2075.487, 2062.402, 1984.201, 1838.201, 1787.159, 684.017, 1582.765, 1444.688, 1301.506, 1293.486, 1238.345, 1148.147, 1081.362, 937.998, 911.105, 894.047

**Match to: gi|39971339, Conserved hypothetical protein [Magnaporthe grisea 70-15] MGG_10690.5**

Score: **166**

Number of mass values searched: **19**

Number of mass values matched: **16**

Sequence Coverage: **51%**

Matched peptides shown in **bold**

1 **MAAPPPIVLD GGTGFLKVGY AGQNFPEHQF PSIVGRPILR** TEEK**GDNILG PDGNEIKIKD**

61  **IMCGDEASAA RTMLQVSYPM ENGIVKKWDD MQHLWDHTFY EKLK**VDPAGR KILLTEPPMN

121 PVKNREQMCE VMFERYNFGG VYVAIQAVLA LYAQGLSSGV VVDSGDGVTH IVPVYESVVL

181 NHLTRRLDVA GRDVTR**NLIA LLLRR**GYALN RTADFETVRQ IKEKLCYVSY DLELDKRLSE

241 DTTVLVESYT LPDGRVIRVG SERFEAPECL FQPHLVDCEQ K**GMGEFLFDT IQAADVDVR**S

301 SLFK**AIVLSG GSSMYPGLPS RLEK**ELKQLW LTKVLGGNPE RLSKFKVRIE DPPRRR**HMVF**

361  **LGGAVLANIM ADKESMWITK QEWEEQGSRV LEKLGPR**S

Protein Length=398

Theoretical MW=44734.7, Predicted pI=6.20

Nominal mass (M_r_)=44714, Calculated pI value=5.92

**Homology (positive) - 95% with Actin-related protein 2 [Botryotinia fuckeliana B05.10]**

**Spot 3. MGG_02610.5**

**m/z ratio**

3376.821, 3234.701, 2975.331, 2703.188, 2008.168, 1948.266, 1441.672, 1328.439, 939.986, 843.928

Match to: **gi|39970287**, **Conserved hypothetical protein [Magnaporthe grisea 70-15]**

Score: **125**

Number of mass values searched: **10**

Number of mass values matched: **9**

Sequence Coverage: **50%**

Matched peptides shown in **bold**

1 **MSSTTEER**QV WQPLHPQVRP RLDPQYVEVH DKLLQYAPPT HTQPWSAAMR **QPSQASVK**TG

61 LDVVPVLTEE VQLERFTILV LTPVTNADDE PRPESGWPVF VWFHGGGFVL GDHSSELDLL

121 TRICATAR**CV VCSVGYRLAP EHPYPAAIED GTDGVRWILS DAQDGGATRF SIDR**NRWAIG

181 GVSAGALLST VTLISLGEAG DLDSGEMARP LR**QVLVVPVV DNTAMPGSGF WSINPHAISP**

241 **SAER**MLWYRR LWLGDADPR**V WSVSVNHASD KQLAYMPPTF TAIGGEDLLA PEGLAFVDQL**

301 **RGAGVDVETM MLPGCPHAIL AFAGCVDKAK DLFDMCVDRI SK**SFGN

Protein Length=346

Theoretical MW=37774.1, Predicted pI=4.76

Nominal mass (M_r_)=37757, Calculated pI value=4.96

**Spot 4. MGG_02252.5**

**m/z ratio**

3091.241, 3041.560, 2274.770, 2235.508, 1994.373, 1799.989, 1652.876, 1170.323, 1107.134, 1095.249, 1092.290, 953.142, 935.114, 899.069, 863.962, 863.934, 849.978, 847.919, 842.987, 823.943

Match to: **gi|39968319, Tetrahydroxynaphthalene reductase [Magnaporthe grisea 70-15]**

Score: **202**

Number of mass values searched: **20**

Number of mass values matched: **18**

Sequence Coverage: **72%**

Matched peptides shown in **bold**

1  **MPAVTQPR**GE SK**YDAIPGPL GPQSASLEGK VALVTGAGR**G IGR**EMAMELG RR**GCK**VIVNY**

61 **ANSTESAEEV VAAIKKNGSD AACVKANVGV VEDIVR**MFEE AVKIFGKLDI VCSNSGVVSF

121 GHVK**DVTPEE FDRVFTINTR GQFFVAR**EAY K**HLEIGGRLI LMGSITGQAK** AVPK**HAVYSG**

181 **SK**GAIETFAR CMAIDMADKK ITVNVVAPGG IK**TDMYHAVC REYIPNGENL SNEEVDEYAA**

241 **SAWSPLHRVG LPIDIARVVC FLASNDGGWV TGKVIGIDGG ACM**

Protein Length=283

Theoretical MW=30047.9, Predicted pI=6.67

Nominal mass (M_r_)= 30034, Calculated pI value= 6.24

**Spot 5. MGG_01084.5**

**m/z ratio**

2915.104, 2653.034, 2373.624, 2350.617, 2092.443, 1844.146, 1664.983, 1646.829, 1587.951,1588.769, 1385.531, 1150.372, 942.075, 895.958

Match to: **gi|39973539** **glyceraldehyde-3-phosphate dehydrogenase [Magnaporthe grisea 70-15]**

Score: **171**

Number of mass values searched: **14**

Number of mass values matched: **13**

Sequence Coverage: **64%**

Matched peptides shown in **bold**

1 MVKCGINGFG RIGRIVFR**NA IEHPDCEIVA VNDPFIEPK**Y AK**YMLEYDST HGRFKGTVEV**

61 **SGSDLVVNGK KVKFYTERDP ANIPWSETGA EYVVESTGVF TTTDK**ASAHL KGGAKK**VIIS**

121  **APSADAPMYV MGVNEKSYDG SASVISNASC TTNCLAPLAK VINDKFGIVE GLMTTVHSYT**

181 **ATQK**TVDGPS AKDWRGGR**GA AQNIIPSSTG AAK**AVGK**VIP ALNGKLTGMS MRVPTANVSV**

241  **VDLTCRLEKG ASYEEIK**AAI KEAADGPLKG ILEYTEDDVV SSDMIGNNAS SIFDAQAGIA

301 LNDKFVKLVS WYDNEWGYSR R**VIDLVTYIS K**VDGGK

Protein Length=336

Theoretical MW =3 5925.0, Predicted pI = 6.91

Nominal mass (M_r_)=35909, Calculated pI value= 6.46

**Spot 6. MGG_09367.5**

**m/z ratio**

3119.600, 2287.763, 2034.378, 1964.347, 1564.789, 1466.639, 1436.579. 1364.469,

1360.521, 1192.369, 1180.383, 1157.237, 1077.195, 1046.184, 1036.188, 804.953

Match to: **gi|39960255**, **Hypothetical protein MGG_09367.5 [Magnaporthe grisea 70-15]**

Score: **214**

Number of mass values searched: **16**

Number of mass values matched: **16**

Sequence Coverage: **62%**

Matched peptides shown in **bold**

1 **MFAASRIQTR** AFSAAARNLS K**VTVLGAAGG IGQPLSLLLK LNPRVTELAL YDIRGGPGVA**

61 **ADISHINTK**S NVK**GYDPTPS GLAAALKGSE VVLIPAGVPR KPGMTRDDLF NTNASIVR**DL

121 AK**ACAESCPE ANILVISNPV NSTVPICAEV FK**ARGVYNPK RLFGVTTLDV VR**ASRFVSEI**

181 **K**GSDPKDENI TVVGGHSGVT IVPLFSQSNH PDLSANDQLV NR**VQFGGDEV VKAKDGAGSA**

241  **TLSMAMAGAR MAESVLR**AAQ GEK**GVIEPTF VDSPLYKDQG IEFFSSK**VEL GPNGVEKILP

301 IGEIDANEQK **LLDACVGDLK** KNIEKGVAFV ASNPGK

Protein Length=336

Theoretical MW=35184.6, Predicted pI=8.31

Nominal mass (M_r_)=35169**,** Calculated pI value=8.26

**Homology (positive) - 94% with malate dehydrogenase, mitochondrial precursor [Chaetomium globosum CBS 148.51]**

**Spot 7. MGG_06561.5**

**m/z ratio**

3226.740, 3065.549, 2973.479, 2929.402, 2699.006, 2447.707, 2306.645, 2077.271, 1776.906, 1340.549, 1262.541, 1107.285, 945.033, 905.054, 848.006

Match to: **gi|145608532, hypothetical protein MGG_06561 [Magnaporthe grisea 70-15]**

Score: **156**

Number of mass values searched: **15**

Number of mass values matched: **13**

Sequence Coverage: **53%**

Matched peptides shown in **bold**

1 MSRYLTRRLA QTTASLRLSS GRRCYSAKAS FDWKDPLNSK **SLLTEEEVAI SETAERYCQE**

61 **KLAPR**VLEAY RNESYDR**AIL SEMGELGLLG ATISGHGCAG VSTVAGALIT R**AVERVDSGY

121 R**SGMSVQSSL VMGGIDYWGS TEQK**ERFLPQ MAAGKLLGAF GLTEPNHGSD PASMETVARP

181 HPTKK**GFYSL SGSK**TWITNS PIADILLVWA KLADGEGPSK IR**GFLVERKN CPPGTLETPA**

241 **IK**NKTGLR**AS LTGMIHLDNC PIPEENMFPT VTGLKGPFTC LNSARFGIAM GVMGALEDCI**

301 **AATR**EYALER SQFGRPLASF QLVQKKLADA ATDAAYGSLA AIQVGRLIDE GK**SVPDMISM**

361 **VKR**ENCDRAL RNAR**TLQEVL GGNAVSDEYR IGRHVANLFV TQTYEGQSDI HSLILGRAVT**

421  **GIQAFV**

Protein Length=426

Theoretical MW=46087.2, Predicted pI=8.37

Nominal mass (M_r_)=46066, Calculated pI value=8.53

**Homology (Positives) - 82% with glutaryl-CoA dehydrogenase [Coccidioides immitis RS]**

**Spot 8. MGG_05861.5**

**m/z ratio**

3080.483, 2917.360, 2683.903, 2416.756, 2130.421, 1713.879, 1659.597, 1599.860, 1311.468, 1303.552, 1238.404, 1206.399, 1121.333, 1071.196, 1030.197, 1016.115, 1007.147, 916.979, 916.985, 873.121, 861.854, 830.926

Match to: **gi|39976425, Predicted protein MGG_05861.5 [Magnaporthe grisea 70-15]**

Score: **182**

Number of mass values searched: **22**

Number of mass values matched: **19**

Sequence Coverage: **59%**

Matched peptides shown in **bold**

1 MTVVRR**NLLH SPSTSPEIKF TPPHKTATGL VQNGR**DHGGK **DTDETPGKDA PPLASDMAAP**

61 **R**DDGILR**TWH** **YSHLKYDPTP PSNHCGTIRR ADHTLHLDGI VDLVVAATPD IPVMTFR**NPH

121 LGK**SAAHAAA YR**ANTRKR**YL SDLLQR**YVES GHVLVFVLPG GNQVVGLAVW SYHNASVDGE

181 LGSGDITVTR **EELAELK**AEV KR**QKRNGGGA ENGDHDAGDA FRDAAFRIYN LR**CQDSLAK**G**

241  **GNEPMMLWLR MLCVHPEHR**G K**GIGAAMVEE GIRLQR**GDGV TKWAALYSSS EAAAKLYRRL

301 GFRDHGGFEI KLGELDNVDG EEKLNVRVMA RKIRQGSEVE LR**DVELINPC KVHIPTAAQL**

361 **EKWGVTDWNN LVVEGLPWDG VVSQIALRR**

Protein Length=389

Theoretical MW=42869.8, Predicted pI=7.79

Nominal mass (M_r_)=42851, Calculated pI value=7.31

**Spot 9. MGG_00604.5**

**m/z ratio**

2783.046, 2649.992, 2156.372, 2030.316, 1703.962, 1585.827, 1469.716, 1346.470, 1294.582, 1073.267, 1042.186, 905.972

Match to: **gi|39974499, Beta-tubulin [Magnaporthe grisea 70-15]**

Score: **126**

Number of mass values searched: **12**

Number of mass values matched: **11**

Sequence Coverage: **36%**

Matched peptides shown in **bold**

1 MREIVHLQTG QCGNQIGAAF WQTISSEHGL DSNGVYNGTS ELQLER**MSVY FNEASGNK**HV

61 PR**AVLVDLEP GTMDAVRAGP FGQLFRPDNF VFGQSGAGNN WAKGHYTEGA ELVDQVLDVV**

121 **RR**EAEGCDCL QGFQITHSLG GGTGAGMGTL LISKIREEFP DR**MMATFSVV PSPK**VSDTVV

181 EPYNATLSVH QLVENSDETF CIDNEALYDI CMRTLKLSNP SYGDLNYLVS AVMSGVTTCL

241 RFPGQLNSDL RKLAVNMVPF PRLHFFMVGF APLTSRGAHS FR**AVTVPELT QQMFDPKNMM**

301  **AASDFRNGRY LTCSAIFR**GK VSMK**EVEDQM RNVQNKNSSY FVEWIPNNIQ TALCSIPPR**G

361 LK**MSSTFIGN STAIQELFKR** VGEQFTAMFR RKAFLHWYTG EGMDEMEFTE AESNMNDLVS

421 EYQQYQDAGV DEEEEEYEEE APLEGEE

Protein Length=447

Theoretical MW=49901.2, Predicted pI=4.51

Nominal mass (M_r_)=49878, Calculated pI value=4.80

**Spot 10. MG03185.4**

**m/z ratio**

3290.885, 2953.305, 2857.240, 2522.669, 2510.790, 2014.277, 1931.307, 1773.991, 1601.832, 1502.765, 1436.538, 1404.620, 1266.442, 1217.425, 1148.316, 1129.311, 1116.318

Match to: **gi|145608354, ATP synthase beta chain, mitochondrial [Magnaporthe grisea 70-15]**

Score: **162**

Number of mass values searched: **17**

Number of mass values matched: **15**

Sequence Coverage: **47%**

Matched peptides shown in **bold**

1 MFKSGLSPIA RAARPASRLA ARPSLRLPKY SLGARFASTQ GVGDGKIHQV IGAVVDVKFD

61 TAKLPPILNA LETTNNNQKL VLEVSQHLGE NVVR**CIAMDG TEGLTR**GAKA K**DTGAPITIP**

121 **VGSGTLGRIM NVTGDPIDER** GPIKTDK**YLP IHAEAPEFVE QSTTAEILVT GIKVVDLLAP**

181  **YARGGKIGLF GGAGVGKTVF IQELINNIAK AHGGYSVFTG VGERTREGND LYHEMQETSV**

241 **IQLDGESKVS LVFGQMNEPP GARARVALTG LTVAEQFR**NE GQDVLLFIDN IFRFTQAGSE

301 VSALLGRIPS AVGYQPTLAV DMGGMQERIT TTQKGSITSV QAVYVPADDL TDPAPATTFA

361 HLDATTVLSR **GISELGIYPA VDPLDSKSRM LDPRIVGQEH YEVATRVQQI LQEYK**SLQDI

421 IAILGMDELS EADKLTVERA RKIQRFLSQP FTVAQVFTGI EGKLVDLKDT IASFK**AILAG**

481  **EGDSLPEGAF YMVGDLASAR** EK**GEKILAEL EK**DA

Protein Length=521

Theoretical MW=55884.4, Predicted pI=5.27

Nominal mass (M_r_)=55022, Calculated pI value=5.42

**Spot 11. MGG_02992.5**

**m/z ratio**

3331.812, 3099.402, 2313.624, 2188.464, 2117.435, 2041.296, 1897.117, 1855.293, 1653.973, 1619.865, 1618.773, 1594.736, 1217.422, 1160.279, 1113.203, 1095.167, 1095.188, 1012.045

Match to: **gi|145609776**, **Hypothetical protein MGG_02992 [Magnaporthe grisea 70-15]**

Score: **124**

Number of mass values searched: **18**

Number of mass values matched: **14**

Sequence Coverage: **41%**

Matched peptides shown in **bold**

1 **MSNFQDPTR**V VPAPSWGLTY RPPAIEDGVD SGLEDDEEEQ PIEPDDPKIF RDGRAPRDPR

61 MQGSARKNLM MFKKLCGDDA LKK**VILATTM** **WDIVPTETAE ARQAELVNTP EFWGYMVEK**G

121 SRICRHHNTI GSARKIIESL IQDR**NTMILE LQNQMVNESR PLQDTAAGME LRK**EFARERK

181 RWERELRETQ ENMEEAIRLR DKESEEALLE LKTEYTERIE RLEREHDKLR **TNAEQLHQER**

241 **IVRFR**NAFKV KEIVRGKGHE EDQGDPLDAP TFSYSMVGGI YCFTGPVTYW SGAWICKYR**N**

301 **HGGNWATR**YN K**TLVELYPNL R**QKIQTSRPW VESWPHWICM GPAPHYILQW NARATAESR**Q**

361 **DAFLPVPVAE VLKKVRVDGV LQAAALGFNT SYVFVDVK**GK ATWDLGGHYP GLKRRLEQHS

421 TDIEYR**NGSM AYSVERCMPN EHFESWLVK**N TELEFGASKD

Protein Length=460

Theoretical MW=53325.2, Predicted pI=6.31

Nominal mass (M_r_)=53301**,** Calculated pI value= 5.99

1. Proteins identified by Matrix Assisted Laser Desorption Ionization- Time of Flight Mass Spectrometry (MALDI-TOF MS). These proteins were differentially expressed on pH scale 4-7.

**Spot 12. MGG_09874.5**

**m/z ratio**

3046.354，2857.169，2646.062，2118.330，2096.332，1941.167，1275.462， 1007.165，1001.072

Match to: **gi|145602787, Hypothetical protein MGG_09874 [Magnaporthe grisea 70-15]**

Score: **120**

Number of mass values searched: **9**

Number of mass values matched: **8**

Sequence Coverage: **68%**

Matched peptides shown in **bold**

1 **MPVDFDKR**EY WHERFSSETK FEWLITSERF MAILEPLLSQ LPK**TSRILQL GSGNSDLHNH**

61 **LRACGFANVT NIDYEPLAIE R**GR**QLEKLAF GDVRMRYLVA DATEIDPTSL CSEGRFDLVV**

121 **DKSTADALSC GGNEAVMDML R**GVK**ECLDAE HGKWVSVSYS EHRFSLDENP FHVDVMHK**VP

181 TPKRLETDPD IFHWCYMLSP

Protein Length=200

Theoretical MW=22951.1, Predicted pI=4.98

Nominal mass (M_r_)=22940, Calculated pI value= 5.15

**Spot 13. MGG_04319.5**

**m/z ratio**

2827.131, 2434.720, 1999.152, 1452.726, 1219.444

Match to: **gi|39944616, Predicted protein [Magnaporthe grisea 70-15]**

Score: **69**

Number of mass values searched: **5**

Number of mass values matched: **4**

Sequence Coverage: **52%**

Matched peptides shown in **bold**

1 **MPGIPLHAFD NLK**AKLKAAF NERKKKK**SSS EKPADPAATA TTAPTPAKTD AAPAAAPAAA**

61  **AVASTPAAAA LDAAKK**DDAT AAPAAAEPAS AAFEPAKPAE PTVTEPAATE AAPAPATSTT

121 TAGAEASAAP APTPAEPAAP AIETDK**AEEK KPQATDAAPA AAPAAPTAAA APVAPTA**

Protein Length=177

Theoretical MW=16803.3, Predicted pI=4.79

Nominal mass (M_r_)= 16797, Calculated pI value=5.06

**Spot 14. MGG_02234**

**m/z ratio**

1933.292, 595.776, 1284.442

Match to: **gi|39968283, Hypothetical protein MGG_02234 [Magnaporthe grisea 70-15]**

Score: **46**

Number of mass values searched: **3**

Number of mass values matched: **3**

Sequence Coverage: **19%**

Matched peptides shown in **bold**

1 MHFPTILVTL AAAVSTTSAI NVRSHSGNTC NGEWSQCSNI AIAYTARIR**G EAYR**DSGCST

61 FAGSELSRGA DWICIPYTSA RPGRR**TGGRW YNSSTR**RRSV GALEGDDNNK TCPAEQYVEE

121 GECRTYVKPD LFGLADGTTF NITGLDEDKV MELEK**LAGTG TAIDAMPAEF QALR**SSEE

Protein Length=178

Theoretical MW=19273.8, Predicted pI=4.85

Nominal mass (M_r_)=19265, Calculated pI value=5.15

**Spot 15. MG08592.4**

**m/z ratio**

3091.325, 2677.959, 2057.332, 1527.559, 1229.344, 998.059

Match to: **gi|39946616, Predicted protein [Magnaporthe grisea 70-15]**

Score: **103**

Number of mass values searched: **6**

Number of mass values matched: **6**

Sequence Coverage: **60%**

Matched peptides shown in **bold**

1 MSEEEK**VVTH EDIWDDSALV NSWNEALEEY KKYHSIHADR AAEATIVPDS QKSGHFPPFF**

61 **AVSTSPGRPL RNAKTETNEP QSPPNGTR**GD GETIQEQAKP TSGCPEGSGV NDQQHGGALS

121 SPISVLGSVK DEGLK**SLLMS WYYAGYYTGL YEGQQQR**DPG KVNPDRR

Protein Length=167

Theoretical MW=18318.7, Predicted pI=4.74

Nominal mass (M_r_)=18311, Calculated pI value=4.98

**Homology (positive) -** 46% with SMN family protein Smn1 [Schizosaccharomyces pombe]

**Spot 16. MGG_06153.5**

**m/z ratio**

2880.265, 2584.810, 2281.697, 1797.09, 1761.945, 1346.578, 1316.376, 1176.299, 1149.215, 830.973

Match to: **gi|39975841, Hypothetical protein MGG_06153.5 [Magnaporthe grisea 70-15]**

Score: **109**

Number of mass values searched: **10**

Number of mass values matched: **9**

Sequence Coverage: **44%**

Matched peptides shown in **bold**

1 MASADERNSR **TVHTAACLII GDEVLGGKTV DTNSNTVAK**W CFELGINLKR IEVIEDDESE

61 IIEAVRRMSD R**YDFVVTSGG IGPTHDDITY QSIAKAFGLD LKLHNDAYER** MKKLSRPHPS

121 QPKFNWDEDS PAKRAKLR**MV ELPTDESR**DM DKQFLFPREE LWVPVCVVNG NVHILPGVPK

181 LFVSLLEGLK PYIVPRLVDP EGKGICR**VMI STPLAESAMA AYLTELAAK**V EPQGVKVGSY

241 PRWNKKR**NTV TLVGK**NK**AFI ESLIPEVTK**N VQGR**VITVEG EDDDPK**DKDE PVSQ

Protein Length=294

Theoretical MW=32851.7, Predicted pI=5.50

Nominal mass (M_r_)**=**32837, Calculated pI value=5.56

**Homology (positive)- molybdopterin binding domain containing protein [Pyrenophora tritici-repentis Pt-1C-BFP] 82%**

**Spot 17. MGG_00867.5**

**m/z ratio**

3458.907, 3110.541, 2573.966, 2176.487, 1881.103, 1600.663, 1479.641, 1462.743, 1431.557, 1173.237

Match to: **gi|39973973, Hypothetical protein MGG_00867 [Magnaporthe grisea 70-15]**

Score: **92**

Number of mass values searched: **10**

Number of mass values matched: **7**

Sequence Coverage: **55%**

Matched peptides shown in **bold**

1 MPTHYKNTRL FGGAIVAEIP AQFADVSKIR **QVPDNQEVFI DKDGFTSIIV EITERVGGPG**

61 **SSAEIDGKAL SVHLEEIVGS DVEGVKVWNT TDTEFTLLGS KIPAYTLIAT QTPK**ADSKRG

121 SSSSPDFTAL ILTLLRLER**E KTDILITVNV PHIKGSYDED EIDLEMGK**QG KLIGDAVEHA

181 ARIWSSFDIL DWKLFNEV

Protein Length=199

Theoretical MW=21812.7, Predicted pI=4.48

Nominal mass (M_r_)=21803, Calculated pI value=4.74

Ran GTPase binding protein Mog1 [Schizosaccharomyces japonicus yFS275]

**Spot 18. MGG_03983.4**

**m/z ratio**

1803.048, 1691.908, 1022.162, 801.894, 784.947

Match to: **gi|39943944, Hypothetical protein MGG_03983.4 [Magnaporthe grisea 70-15]**

Score: **52**

Number of mass values searched: **5**

Number of mass values matched: **4**

Sequence Coverage: **22%**

Matched peptides shown in **bold**

1 MGDK**NLNALL K**WSIENTNTD GSAPAAGADQ QQELQRPDPE VLAALFGGPS EAELMKAAMD

61 VITSTEPDVT LDNKLIAFDN FEQLIESLDN ANNLSKLSLW TPLLGLLDSD HPDLRRMAAW

121 CIGTAVQNNE PCQERLLALG GLPSLVKLAT AEDQREDVRR K**VVYALSSAG RNYQPAMDVI**

181 **VEEVGK**QGGK SDKVDATNMD **AVDAVIDLLK** AGIPKKS

Protein Length=217

MW=23408.1, Predicted pI=4.26

Nominal mass (M_r_)=23398, Calculated pI value=4.49

**Homology (positive)- Hsp70 nucleotide exchange factor fes1 [Aspergillus oryzae] 72%**

**Spot 19. MGG_06348.5**

**m/z ratio**

3493.956, 3367.838, 2546.802, 2206.423, 1685.860, 1671.886, 1471.667, 995.096

Match to: **gi|39976891, Conserved hypothetical protein [Magnaporthe grisea 70-15]**

Score: **75**

Number of mass values searched: **8**

Number of mass values matched: **6**

Sequence Coverage: **45%**

Matched peptides shown in **bold**

1 **MATNITWHDG LTRR**ERNQLR GQR**GFTIWFT GLSASGK**STV ATALEQHLLH LGLAAYRLDG

61 DNVRFGLNKD LGFSEKDRNE NIRRIAEVAK LFADSSVVAL TSFISPYRAD RQTAR**ELHAQ**

121  **AAQKGDEPLE FVEVYVDIPI EVAEQR**DPKG LYKKARAGEI K**EFTGISAPY EAPENPEITI**

181 **KTHENSVEEC VAQIVK**WLQD KGLVKATP

Protein Length=208

Theoretical MW=23288.2, Predicted pI=6.69

Nominal mass (M_r_)=23278, Calculated pI value=6.24

**Homology (positive)- 80% with adenylylsulfate kinase [Aspergillus nidulans FGSC A4]**

**Spot 20. MGG_10637.5**

**m/z ratio**

2294.686, 2124.427, 1965.229, 1890.132, 1727.836, 1704.894, 1687.682, 1526.805, 1460.722, 1425.694, 1316.527, 1068.294, 855.991, 847.833

Match to: **gi|39970057**, **Predicted protein [Magnaporthe grisea 70-15]**

Score: **144**

Number of mass values searched: **14**

Number of mass values matched: **12**

Sequence Coverage: **54%**

Matched peptides shown in **bold**

1  **MSFVAAESPP IHIK**SSPGST SGTEGSNSDS DSDGSDIGDI PYFLPDTDIT APSNNLSVDN

61 FDQDILAQFL GDDGDSQDPV NDTVDEPDYL IR**SDIEDPNE INIDVVRDIL SQMPSNLAKN**

121  **GATTAMLGMQ PLRSCVIHPV WK**SRKIIWKR LDNVEAVLMQ IVGTENPIAC KRCTDNR**GPF**

181 **VGWYSVPGVH NGGCGCCK**YN AQTAKCSLYK GSKRPRRTSQ RPLKRRKR**GN EASNTRDAGP**

241 **SVPAAQPLAI QNRNPVSLSP AYLVQPAPQQ RVLQVPIPAE LGWEDAMTLR RALQEMAEQL**

301 **VFSPQGNK**VV RRVLR**EFSGA LEDFASEEQE**

Protein Length=330

Theoretical MW=36158.0, Predicted pI=4.87

Nominal mass (M_r_)=36142, Calculated pI value=5.12

**Spot 21. MGG_08809.5**

**m/z ratio**

3216.618, 3078.475, 2365.663, 2321.509, 2126.593, 1784.079, 1360.487, 1302.494, 1235.413

Match to: **gi|145601361, Hypothetical protein MGG_08809 [Magnaporthe grisea 70-15]**

Score: **92**

Number of mass values searched: **9**

Number of mass values matched: **7**

Sequence Coverage: **45%**

Matched peptides shown in **bold**

1 MK**AEEVVVEE AFVEETIIEE TR**TQDGTISP EAEAESALES EDPDATPWYL EVEPPRHPTL

61 VPHDIPLPPT PADSPALVGP LIKFVAEDLG VDDFNVLDLR **TVEPPPALGP KLIMMFGTAR**

121 SER**HLHVSAD RLVRWLRGHG VTAHADGLLG R**NELKTKMRR RARK**AKLMGD TAWDKGDDGI**

181 **STGWICVNLG TIGWKPMAPE DEVVR**TDAEG RITGFGTPIE GTTLVVQLMT EGKREELGLE

241 RLWNGVLKKS DRDR**AEIAAA QNVEDVPYFP PHIKR**GKRGR RA

Protein Length=282

Theoretical MW=31216.9, Predicted pI=4.95

Nominal mass (M_r_)=31203, Calculated pI value= 5.18

**Homology (positive)-40% with Atp25p [Saccharomyces cerevisiae S288c]**

**Spot 22. MGG_10674.5**

**m/z ratio**

3075.295, 3040.364, 2488.816, 2297.366, 2132.169, 2118.514, 2117.154, 1887.962, 1544.805, 1510.523, 1401.630, 1122.282, 1034.982, 862.045, 834.964, 812.963

Match to: **gi|145604182, Hypothetical protein MGG_10674 [Magnaporthe grisea 70-15]**

Score: **141**

Number of mass values searched: **16**

Number of mass values matched: **13**

Sequence Coverage: **44%**

Matched peptides shown in **bold**

1 **MPAIIETDLP SSK**ADKKSKK NKEKKRPR**ED** **DEATEAR**NNK RSKSEAPTAD EAEDGQHVNG

61 GEKKKHKKSR KSK**QTETEDV EVEPEQVEEA R**AKKEKKQKK KK**DKTTNGED ASEEVVPESP**

121 **VK**STKKSKKV KSEVEAAPET IVDNAATEEA APEKKKKKRS RRKSDR**AAEE DVEEEYGAAK**

181 TKK**SDSEALP PDSPASQGFA LDLMDVDSPS VAR**SAKPDIL QPAHAPSNPE FPFATQIVSL

241 YVPLFPIGFD QPLTK**VAEQH LKPLLNHYSP LMKGVLLDYR HVTLGELPTR** ADPRNPPTDR

301 **TPTLLSCKDE YAVGFGWLTA EVYLFVPRR**G AWMEGVVNLQ SEGHLGVVCW NRFNASIEAN

361 R**VPEGWKFID VVQK**AEDAKK AKFRNAGKKI NLEEAEGEGD EEAE

Protein Length=404

Theoretical MW=44963.4, Predicted pI=6.66

Nominal mass (M_r_)=44944, Calculated pI value=6.29

**Spot 23. MGG_10684.5**

**m/z ratio**

2973.361, 2800.178, 2697.055, 2186.349, 2094.457, 1815.001, 1441.595, 1311.412, 1196.364, 972.160, 945.076

Match to: **gi|39971327, Hypothetical protein MGG_10684 [Magnaporthe grisea 70-15]**

Score: **106**

Number of mass values searched: **11**

Number of mass values matched: **9**

Sequence Coverage: **37%**

Matched peptides shown in **bold**

1 MSDLDRFCWQ YLQLEQRLDY PPRELIR**TEE CQSQLFARLF APGALPHPPP HR**YQLRVLKE

61 LIGRIEASIE DWDEHGISDD LATALTALMS QPTPSEATAA QQK**SYVTYHL SLLDQPSSSS**

121 **SSKSITLLEN RHLIAASGTT GLRTWEAALH LGQYLCLHAD SLVSGRR**VLE LGAGTGYLSM

181 LCAAHLGAAH VVASDGSDDV VANLPEGAFL NGLQHEGQAA AAAAER**AAVQ PMDLK**WGHAL

241 VGTEDARWNG GEPVHLVLGA DVTYDATLAA PLLGTIAELV GMFPDVQVLI AATER**NSATY**

301 **AAFLDAVAKS ADVALLEEVA LAVDGSR**GPF FPDNVPIHIC RLGRRV

Protein Length=346

Theoretical MW=37209.4, Predicted pI=4.85

Nominal mass (M_r_)=37193**,** Calculated pI value=5.03

**Spot 24. MGG_07401.5**

**m/z ratio**

3207.575, 3051.459, 2890.056, 2820.110, 1993.380, 1925.220, 1824.143, 1210.304, 1159.401, 1029.258, 993.166, 850.963

Match to: **gi|145616429** **Hypothetical protein MGG_07401 [Magnaporthe grisea 70-15]**

Score: **140**

Number of mass values searched: **12**

Number of mass values matched: **11**

Sequence Coverage: **49%**

Matched peptides shown in **bold**

1 **MAQTSTALVL YQPYPNKSLA EYIR**AVEESH NAYTAQLSPT GQLPALMALP VELFMQIYDL

61 LTVESQAMLA VTCKSLYR**SM FPHVAIK**QVK HKGGMLRCIE R**QMPQQYYCP VCVKLHRWHL**

121  **DDNGRPVFPT CDDMTPSCGV TRWLFPSDPG PYAEGSPETV DYEVDYR**TAR LAVR**AAALGP**

181  **SFGPPLSILE KRAAWQPCPD MADQHGPLLN AVAVGYHYEP RVVDGVLHVR** ATYVVRDNSG

241 SPDLWNLWAK KPDLGPWICP GRK**VFAEFEG QAGR**CLCFED VPGTDVDAES SSDVEEEVVE

301 CSCAK**TLAEV CALAIR**TVSR CECAAWASQY CGTEWEITNL VVVVVVVFNA GLGPAH

Protein Length=356

Theoretical MW=39325.0, Predicted pI=5.08

Nominal mass (M_r_)=39306, Calculated pI value=5.23

**Spot 25. MGG_06099.5**

**m/z ratio**

3198.637, 2908.198, 2666.878, 1925.288, 1863.108, 1833.987, 1671.895, 1646.872, 1530.750, 1484.617, 1386.580, 1159.389, 984.116, 951.042

Match to: **gi|145603364, Serine/threonine protein phosphatase PP2A catalytic subunit [Magnaporthe grisea 70-15]**

Score: **195**

Number of mass values searched: **14**

Number of mass values matched: **14**

Sequence Coverage: **67%**

Matched peptides shown in **bold**

1 MEDVGR**VPSE LAPQPSSEPT TIPTLDGWIE SLMACKQLVE ADVQRLCEKA** **REVLQDESNV**

61 **QPVK**CPVTVC GDIHGQFHDL MELFKIGGPN PDTNYLFMGD YVDR**GYYSVE TVTLLVALKI**

121 **RYPQRITILR** GNHESRQITQ VYGFYDECLR KYGNANVWKY FTDLFDYLPL TALIDNQIFC

181 LHGGLSPSID TLDNIRALDR IQEVPHEGPM CDLLWSDPDD RCGWGISPRG AGYTFGQDIS

241  **EAFNHNNGLT LIARAHQLVM EGYNWSQDRN VVTIFSAPNY CYRCGNQAAI MEIDEHLKYT**

301 **FLQFDPCPRA GEPLVSRR**TP DYFL

Protein Length=324

Theoretical MW=36856.3, Predicted pI=4.59

Nominal mass (M_r_)=36839, Calculated pI value=4.81

**Spot 26. MGG_04684.5**

**m/z ratio**

3332.940, 2888.319, 2373.723, 2178.465, 2177.388, 1971.060, 1839.139, 1817.256, 1799.102, 1375.656, 1356.487, 1194.261, 1180.380, 1059.219, 973.089, 965.112, 898.964, 860.912, 859.928, 806.950

Match to: **gi|39945404, Hypothetical protein MGG_04684 [Magnaporthe grisea 70-15]**

Score: **194**

Number of mass values searched: **20**

Number of mass values matched: **19**

Sequence Coverage: **50%**

Matched peptides shown in **bold**

1 **MQSFVVIAGL LAAAYFVHRL LYGTDTPHIK GLPEVPGLPL FGSLIELGDN HAK**VAQRWAE

61 K**YGPVFQVR**M GNRRIVFANT FASVRHLWIT NQSALISRPK LHTFHTVVSS SQGFTIGTSP

121 WDDSCRRRRK **AAATALNRPA VQSYMPLIDL ESTVSIRELL QDSRGGAVDL DPR**AYWQR**YA**

181 **LNTSLTLNYG YRIDGDK**DDE LLREITDVER GVSNFR**STSN NWQDYVPLLR LFPK**MSREAV

241 EFRERR**DVYM DRLLR**GLKER IER**GMDKPCI SGNILKDPEA KLNEAEIK**SI GLTMVSAGLD

301 TVPGNLIMGI AYLASPAGQE IQQR**AYAEIL KTYPANDAWT RCLDEEKIPY ITALYKEILR**

361 **FFTVIPICLP R**VSVKDVEWE GVTMPAGTTF YMNAYAADYD AAHFSDPYVF NPDR**YLEDVE**

421 **GTPHYGYGAG SRMCAGSHLA NR**ELYTAFVR LISAFEFVPP ADPADEAVLD CLEANAIPTS

481 LTMEPKYFKV GFRPRDR**DVL DGWIR**ESEER TRHLM

Protein Length=515

Theoretical MW=58310.4, Predicted pI=6.52

Nominal mass (M_r_)=58284, Calculated pI value= 6.12

**Homology (positive) – 84% with Cytochrome P450 phenylacetate 2-hydroxylase, putative [Aspergillus fumigatus Af293]**

**Spot 27. MG02540.4**

**m/z ratio**

3280.782, 3056.387, 3010.476, 2894.175, 1944.121, 1906.217, 1840.045, 1806.986, 1764.946, 1431.711, 1424.583, 1398.678, 1305.402, 1084.187, 1005.174, 994.034, 935.040, 872.970

Match to: **gi|39970147, Conserved hypothetical protein [Magnaporthe grisea 70-15]**

Score: **125**

Number of mass values searched: **18**

Number of mass values matched: **13**

Sequence Coverage: **44%**

Matched peptides shown in **bold**

1 MASMRAVATL ARPAAATLRP QTLAALIPSS ASASTTTSSS AINTSTTPSS RLRRSIPSRR

61 **QISTTAPR**AS SRAPKPK**HPA GFVAPDQAAL DELRASVQEF VRAEIPEEVA AR**TDKENEFP

121 ADMWGKLGDM GLLGVAAEES VGGLAMGYQA HCIVMEELSR **ASASIALSYA AHSQLCVNQL**

181 **QLNGNPEQK**Q RFMPDLIAGR K**VGALAMSES GSGSDVVSMR** TRATR**TADGS GWLLNGSKMW**

241  **ITNGPDADVI IVYAKSDTEC QPSKGITAFI VER**QPGLQCL RKLDKMGMRG SNTGELVLEN

301 VIVPDENVLG EVNAGVRVLM EGLDLERLVL SAGPLGIMQA ALDVALPYAH ARQQFGRPIA

361 EFQLVQGRLA DMYTKLQVSR AYTYATARRI DESGEIK**TAD CAGAILYAAE RATEVALDCI**

421 **QVLGGMGYTE DMPASRLLR**D AKLYEIGAGT SEVRRMVIGR YFNREYSLNQ

Protein Length=470

MW=50462.3, Predicted pI=7.0

Nominal mass (M_r_)=50440, Calculated pI value=6.93

**gi|59803160, Isovaleryl-CoA dehydrogenase-like protein [Magnaporthe grisea 70-15]**

**Spot 28. MG03116.4**

**m/z ratio**

3277.701, 3242.621, 2510.821, 1876.161, 1772.021, 1713.858, 1435.634, 1348.640, 1346.600, 1171.308, 1076.075, 946.107, 860.915, 800.005

Match to: **gi|39942072, Hypothetical protein MGG_03116 [Magnaporthe grisea 70-15]**

Score: **126**

Number of mass values searched: **14**

Number of mass values matched: **12**

Sequence Coverage: **36%**

Matched peptides shown in **bold**

1 **MLCSISGEAP QEPVVSTK**SG NVYEKRLIEK **YIDEHGKEPG SDTDLDKEDL LPIQTSR**VVR

61 PRPAALTSIP ALLSTFQNEW DSLALETYNL QQQLQRTR**EE LANALYQHDA AIRVIARLTK**

121 ERNEARDALA KVTVSAPAPA AANAGDSAMA IDSELPQDVA EHVDETAQML SKSRKKRPTP

181 PTWASPEDIS SLEKVASDSL HPLTQATSLG VDGGYAAVGG FSGDLAIYSL EAAKLER**TLN**

241 **VGEPVTAVLW SGTK**VILGTT KGAVKVYDSG SEVASFSEHA GGVTGLAIHP GSDIVASVGA

301 DR**GVVLYQLS TLKK**VFQTR**T DASLTMCAFH PDGALVAAGT TSGDIKLFHT KTLEEAASFP**

361 **LGAPVQAVAF SENGYWFAAV AKAQTAVTIM DLRK**SGDAAK VKTLDVGTGV LAALAWDYTG

421 RFLAAAGASG VSVQGYNKSS K**AWTSLLQK**G DAAGAVALAW GPDAKKLVAI KPDGVLEMLG

481 VPSAAEEEAP AAEA

Protein Length=494

Theoretical MW=51759.6, Predicted pI=4.95

Nominal mass (M_r_)=51738, Calculated pI value=5.17

Cell cycle control protein (Cwf8), putative [Talaromyces stipitatus ATCC 10500]

**Spot 29. MG04827.4**

**m/z ratio**

3349.904, 2546.773, 2329.713, 2301.481, 2064.442, 1935.244, 1917.201, 1851.981, 1763.993, 1715.976, 1555.847, 1518.848, 1325.562, 1294.471, 1266.322, 1260.278, 1188.335, 1048.107, 1013.197, 922.004, 900.936, 812.917

Match to: **gi|145614390**, **Hypothetical protein MGG_04827 [Magnaporthe grisea 70-15]**

Score: **179**

Number of mass values searched: **22**

Number of mass values matched: **18**

Sequence Coverage: **55%**

Matched peptides shown in **bold**

1  **MALDPPTYLA SLQNNIRQRP IPWDGAVRTG VLTEEQLAKT RAVEKPNR**DA RK**QTVEADLD**

61  **GYR**ALFVGEP GRPSVLESSN KPIVQYLLVL LSDLLDCVPT LSKALFKDSD PYR**QLLPLLA**

121  **QSSGPEDPIP LLTSHVLVNL IAGSRDESDL TLQKALPVIL SYLSTLTKSS DAGLQDIGVQ**

181 **EYSTVLFGSK** SR**SQFWAQRS ETVAPLINIL RAAAGSSNGD ANGIR**SGSMR GGPVEGSIGG

241 GVGLQLLYHV LLVMWQLSFE AEDVGCDLDD LLPVASFARL PSLLSNITSR QLTDPDLQED

301 LQSLKDMMDE YTATK**TTFDE YVAEVTNGHL R**WSPPHRSQT FWAENARRIL DENNAGVVR**Q**

361 **LAEIMKKPWD NDKQVLAIAC NDIGALVR**EV PEKR**GQLERL GLKTRIMELM GEADENVR**WE

421 SLKALGGWLK **YSFENNK**

Protein Length=437

Theoretical MW=48288.2, Predicted pI=4.80

Nominal mass (M_r_)=48267, Calculated pI value=5.05

**Homology (positive) – 68% with vacuolar ATP synthase subunit H, putative [*Aspergillus fumigatus* Af293]**

**Spot 30. MGG_01759.5**

**m/z ratio**

3011.171, 2729.666, 2649.488, 2643.029, 2142.518, 1959.200, 1892.126, 1797.090, 1773.157, 1622.875, 1614.859, 1593.766, 1565.827, 1349.452, 1216.225, 1142.226, 1124.266, 1039.152, 967.085, 937.044, 913.985, 905.027

Match to:  **gi|302413776 DNA replication licensing factor mcm3 [Verticillium albo-atrum VaMs.102]**

Score: **151**

Number of mass values searched: **22**

Number of mass values matched: **17**

Sequence Coverage: **45%**

Matched peptides shown in **bold**

1 **MVGDPSTAKS QLLRFVLNTA PLAIATTGRG SSGVGLTAAV TSDK**ETGERR LEAGAMVMAD

61 R**GVVCIDEFD K**MSDIDR**VAI HEVMEQQTVT IAKAGIHTSL NAR**CSVIAAA NPIFGQYDPH

121 KDPHKNIALP DSLLSRFDLL FVVTDDIEDA RDR**QVSEHVL R**MHRYR**QPGT EEGAVVRENT**

181 **QQSLGVGMQS QSNDSQRPTD VFEKYDSMLH AGVTVTSGR**G ANKKPEVVSI PFMK**KYIQYA**

241 **K**TRIRPVLTQ EASDRIADIY VGLR**NDEVEG NQRR**TSPMTV R**TLETIIRLA TAHAR**SRLSN

301 RVEER**DAAAA ESILRFALFK** EVVEAESRKK RRK**TQNVEYA SSSEDDSSDE DDDGDLR**STA

361 RASTSRSARG TARTRRGAVN ANGRR**ADTSG APVDADDDDE DQEPEASAPR R**SNRSGRGAV

421 ASSSQSQSQV SYASSVPASQ LETQEEDDED AEADGLADEA AALNIGQDDG PEITASRLGA

481 FRQALGQLQS TDLFEDDTAS LDNVVTAVNA KIGSRSGGAF SKDEAVKALR K**MDEANQVM**

Protein Length=539

Theoretical MW=58471.8, Predicted pI=4.84

Nominal mass (M_r_)=58447, Calculated pI value=5.07

**Spot 31. MG00747.4**

**m/z ratio**

3174.107, 3117.300, 2514.651, 2469.900, 2345.681, 2034.197, 1953.223, 1842.933, 1307.433, 1184.371, 1155.323, 883.026, 805.928

Match to: **gi|39974213, Hypothetical protein MGG_00747 [Magnaporthe grisea 70-15]**

Score: **143**

Number of mass values searched: **13**

Number of mass values matched: **12**

Sequence Coverage: **42%**

Matched peptides shown in **bold**

1 MAPLTGPVTT GLQIFAIPGV VLLIAFLGYA SQWVFHTPDL QPGPLSK**NQI ITFNLLLLNL**

61  **WWNYYKACTV PPGTYTHARP PSGDDDVDDK** TKGKKQEDER **QKPMTPAAGQ R**WCRK**CDAPK**

121 **PPR**AHHCRHC RRCIPKMDHH CPWTGNCVSM QTFPYFYRFL VSTNLALWYL SRLLYLRFRA

181 LFDERNLPAY LGPSPTRIAL LTVVAIACAL VSLALGILLT TTTK**AWLFNR TMIEDWEVER**

241 HESLVGRQAR **GGSTWWASEE EEGGEESSSE ETTTPSNGQK** TLRR**VEFPYD IGIWNNMCQA**

301 **MGTRNFLAWF WPFAGGPTVS RTAGSGVGWD WEENGFNAQA GMWPPPDPDK** QRRGHWR**GNA**

361 DTAVKLPTYA TPEEEKAAFK ARQERDQRLR RRRQLMEAQQ GSGIFAELEE DEELEDLFPE

421 QRKREKYEK**G LDGEPGWTNA DGER**LRDFGV DEDSDDVVED DIVPHNADFD HDEDEDVPLG

481 ELLRRRRVAS RNPDT

Protein Length=495

Theoretical MW=56487.7, Predicted pI=5.41

Nominal mass (M_r_)=56462, Calculated pI value=5.47

DHHC zinc finger domain containing protein [Coccidioides posadasii C735 delta SOWgp]

**Note:**

1. Protein score is -10*Log(P), where P is the probability that the observed match is a random event. Protein scores greater than **68** are significant (p<0.05).

2. Cleavage by Trypsin: cuts C-term side of KR unless next residue is P.

3. Unmatched mass to charge ratios are highlighted in blue shading.
