# Supplementary material for: Proteomic changes associated with deletion of the Magnaporthe oryzae conidial morphology-regulating gene COM1
Source: Biol Direct. 2010 Nov 2;5:61. doi: 10.1186/1745-6150-5-61 (PMC2989938; doi:10.1186/1745-6150-5-61)
Supplement: Additional file 4 — Alignment of the predicted amino acid sequence of MGG_06099.6 (PP2Ac-1), MGG_01690.6 (PP2Ac-2) and MGG_01528.6 (PP2Ac-3) using ClustalW algorithm. [file 1745-6150-5-61-S4.DOC]

MGG_06099.6 MDTNMEDVGRVPSELAPQPSSEPTTIPTLDGWIESLMACKQLVEADVQRLCEKAREVLQD

MGG_01528.6 -------------------------MSDLDEAIAQLRACRPIPEPQVRELCLKAREILLE

MGG_01690.6 -------------------MPGLPASVDLDECISRLYKKELLAESVIEAICAKTKELLMR

** * * . : *. :. :* *::*:*

MGG_06099.6 ESNVQPVKCPVTVCGDIHGQFHDLMELFKIGGPNPDTNYLFMGDYVDRGYYSVETVTLLV

MGG_01528.6 EGNVVTVTAPVTICGDIHGQFHDLMELFRVGGDVPDTNYLFMGDFVDRGFYSLESFLLLL

MGG_01690.6 ESNVVHVRAPVTVVGDIHGQFFDLIEIFKIGGWCPDTNYLFLGDYVDRGMFSVETISLLV

*.** * .***: *******.**:*:*::** *******:**:**** :*:*:. **:

MGG_06099.6 ALKIRYPQRITILRGNHESRQITQVYGFYDECLRKYGNANVWKYFTDLFDYLPLTALID-

MGG_01528.6 CLKVRYPDRMTLIRGNHESRQITTVYGFYDECLRKYGSANVWRYCCDVFDYLALGAIVLG

MGG_01690.6 CLKLRYPNRVHLIRGNHESRGVTQSYGFYTECSRKYGNANVWHHFTDMFDFLTLSVVIN-

.**:***:*: ::******* :* **** ** ****.****:: *:**:*.* .::

MGG_06099.6 ------------------------------------------------------------

MGG_01528.6 ASHTLGPSKNPAEDQAGPEYEVEVCSNTGDVVQRFLRKPRENMIGSQGSTHGGSSPPPEA

MGG_01690.6 ------------------------------------------------------------

MGG_06099.6 --------------------------------NQIFCLHGGLSPSIDTLDNIRALDRIQE

MGG_01528.6 QGGGANSSVGPMTGPPGSGASGSSGGSLGNPAGAVFCVHGGLSPLVDSIDKIRLLDRKQE

MGG_01690.6 --------------------------------DQIFCVHGGLSPSIHSIDQIKIIDRFRE

. :**:****** :.::*:*: :** :*

MGG_06099.6 VPHEGPMCDLLWSDPD-DRCGWGISPRGAGYTFGQDISEAFNHNNGLTLIARAHQLVMEG

MGG_01528.6 VPHDGAMCDLLWSDPD-DIAGWGLSPRGAGFLFGPDATKEFNYKNDLSLIARAHQLVMEG

MGG_01690.6 IPHEGPMADLVWSDPDPERDEFSLSPRGAGYTFGAQVVKKFLAVNGMSHILRAHQLCQEG

:**:*.*.**:***** : :.:******: ** : : * *.:: * ***** **

MGG_06099.6 YNWSQDRNVVTIFSAPNYCYRCGNQAAIMEIDEHLKYTFLQFDPCPRAG-----------

MGG_01528.6 FKEMFDASIVTVWSAPNYCYRCGNVAALLELSEDESGLGVFARSNGEVGRSDGGFSRRNE

MGG_01690.6 YQVLYDDRLSTVWSAPNYCYRCGNMASVLEVSDTGERFFNVFAAAPENDQ----------

:: * : *::*********** *:::*:.: . . . .

MGG_06099.6 ------------EPLVSRRTPDYFL--------------------

MGG_01528.6 ARGVMSEQEVTNRPIGPARRYRVFLAAPQDSRGMPAKKPVADYFL

MGG_01690.6 ---HKDIQPGGEKSADSSALPDYFL--------------------

.. . **
